# Using Lateral Flow Devices to Enhance Molecular Epidemiology and Evaluating the Rabies Surveillance System on Unguja Island, Zanzibar

**DOI:** 10.3390/v18080836

**Published:** 2026-07-29

**Authors:** Cayla Bosch, Ali Z. Moh’D, Ramadhan J. Ramadhan, Andre Coetzer, Ayla J. Malan, Louis H. Nel, Nicolette Wright

**Affiliations:** 1 Department of Biochemistry, Genetics and Microbiology, Faculty of Natural and Agricultural Sciences, University of Pretoria, Pretoria 0002, South Africa; cayla.bosch@tuks.co.za (C.B.); ayla.malan@tuks.co.za (A.J.M.); louis.nel@up.ac.za (L.H.N.); 2 Department of Livestock Development, Ministry of Agriculture, Irrigation, Natural Resources and Livestock, Maruhubi, Zanzibar P.O. Box 159, Tanzania; azahrahn209@gmail.com (A.Z.M.); ramaramajuma@hotmail.com (R.J.R.); 3 Global Alliance for Rabies Control, Manhattan, KS 66502, USA; andre.coetzer@rabiesalliance.org

**Keywords:** rabies, molecular epidemiology, surveillance, lateral flow device, spacio-temporal analysis, Unguja Island

## Abstract

Dog rabies remains a neglected tropical disease in many resource-limited endemic regions, including Unguja Island, Zanzibar. Classically, in such resource-limited settings, an underestimation of the impact of rabies, which directly correlates with insufficient diagnostic capabilities and surveillance efforts, leads to poor investment in effective rabies control. This study combined molecular epidemiology with retrospective analyses of surveillance data from 2016 to 2024 to better understand the transmission dynamics and assess the effectiveness and reliability of the rabies surveillance system on Unguja Island. Towards this goal, rabies RNA was extracted from used lateral flow devices for further molecular phylogenetic analyses of the partial nucleoprotein gene. From this analysis, we were able to conclude that the rabies lyssaviruses presently circulating on Unguja Island represent a distinct endemic cycle in comparison to rabies viruses from elsewhere in Tanzania. In addition, space–time analysis and the evaluation of testing rates identified high-risk rabies areas with particularly inadequate surveillance. These findings allow better understandings of the disease’s impact, which will support improved surveillance and the implementation of targeted control strategies, and will pave the way for, hopefully, the elimination of dog rabies in the longer run.

## 1. Introduction

Rabies has a global impact, especially in resource-limited endemic regions that do not have the capacity to control the disease [1]. The single most significant reservoir for rabies virus (RABV) is the domestic dog (*Canis lupus familiaris*) [2]. Only a few countries have successfully eliminated canine-mediated rabies through mass vaccination programs. Based on a previous study by Hampson et al. [2], Africa and Asia have the highest rabies burden, reporting an estimated 59,000 human rabies deaths each year, although a lack of adequate surveillance systems suggests that these human and animal rabies numbers could be underestimated [2].

Despite being a preventable disease through pre-exposure vaccination and the timely administration of post-exposure prophylaxis for humans, rabies remains a burden in endemic regions, which usually have limited resources due to the “cycle of neglect” [3,4,5]. To break this cycle of neglect, effective surveillance systems are needed to obtain accurate data and raise awareness. Unfortunately, rabies surveillance across Africa is considered inadequate and ineffective [6]. It is important to evaluate rabies surveillance systems to identify gaps, which will allow better case detection and the implementation of interventions such as targeted dog vaccinations. In a recent study, Malan et al. [7] evaluated the robustness of the national rabies surveillance system in South Africa (SA). This study used space–time analysis to first identify high-risk rabies areas. Secondly, testing rates were calculated using the average number of samples submitted, which allowed the authors to determine whether the surveillance data across different species groups were adequate and reliable. These testing rates were then combined with the space–time analysis to determine high-risk rabies areas. By identifying high-risk rabies areas with poor surveillance, resources can be allocated to increase surveillance to obtain a more accurate estimate of the rabies burden, so that appropriate control strategies such as animal vaccination can be implemented to prevent the further spread of rabies.

On Unguja Island (the main island of the Zanzibar archipelago), rabies was first recorded in 1921, and then eliminated, only to be reintroduced to Unguja Island in 1991, and to Pemba Island, also part of the Zanzibar archipelago, in 1998 [8]. The re-introduction of rabies to Unguja and Pemba Island was due to a lack of appropriate control measures, which resulted from limited financial resources, a lack of personnel in the medical and veterinary sectors, and poor surveillance due to inadequate diagnostic facilities [8]. While dogs are the main rabies reservoir, they also face other diseases such as canine distemper, helminthiasis and external parasites, and issues such as poor nutrition [8]. From 2009 to 2015, World Animal Protection (WAP) collaborated with the Revolutionary Government of Zanzibar to implement annual dog vaccinations, which led to a steady decline in rabies cases [9,10]. In 2016, the Global Alliance for Rabies Control (GARC) assisted in establishing laboratory-based passive surveillance using a direct rapid immunohistochemical test (DRIT) for rabies diagnosis, and then implemented strategic vaccination in the areas where rabies cases have been detected and extending the area outwards to try and interrupt further RABV transmission [9,11]. In order to shift Unguja Island to a more active surveillance approach, as suspected rabies samples were mainly submitted through passive surveillance, as described above, the Rapid In-field Diagnosis and Epidemiology of Rabies (RAIDER) toolkit was implemented in 2022. This toolkit uses in-field sample collection and lateral flow devices (LFDs) for the rapid diagnostic screening of rabies cases outside the laboratory, while the Rabies Case Surveillance (RCS) tool is utilized for real-time data capturing and reporting [12]. A recent study by Moh’d et al. [13] evaluated the impact of the RAIDER toolkit’s implementation on the active surveillance program in Zanzibar from 2022 to 2023. The authors found that there was a four-fold increase in the number of samples submitted for diagnostic confirmation per month. In addition, the number of shehias (wards) that submitted samples for diagnostic confirmation per year increased three-fold, and a sustained increase in rabies cases that were diagnosed was observed [13,14]. The real-time data gained from implementing the RAIDER toolkit allowed the government to respond quickly by initiating targeted dog vaccinations in areas where rabies cases were diagnosed [13,14].

While LFDs make it possible to have Point of Care (PoC) testing for suspected rabies cases, leading to an increase in rabies surveillance in animals and allowing the administration of post-exposure prophylaxis (PEP) to exposed individuals and targeted animal vaccination, the subsequent molecular analyses of these LFDs will create the possibility for further enhanced surveillance and the genetic characterization of RABVs, such as determining transmission dynamics and tracking endemic cycles in different host populations [15,16,17,18,19]. The use of PoC testing to enhance animal surveillance, supplemented with genomic surveillance using molecular analysis, will allow the local authorities to implement immediate animal vaccination and give better insights into RABV transmission in the geographic area [15,17,20].

Therefore, this study investigated the use of rabies-positive LFDs from the study by Moh’d et al. [13] for further molecular analysis to enhance genomic surveillance, which helped determine the transmission dynamics of RABV in Unguja Island. Furthermore, rabies surveillance data are reported through Unguja Island’s established rabies surveillance system. This study therefore evaluated whether the existing rabies data on Unguja Island were sufficient to analyze whether the rabies surveillance approach is reliable, and to identify high-risk rabies areas with inadequate surveillance that needs to be improved.

## 2. Materials and Methods

### 2.1. Project Site

**Figure 1:**
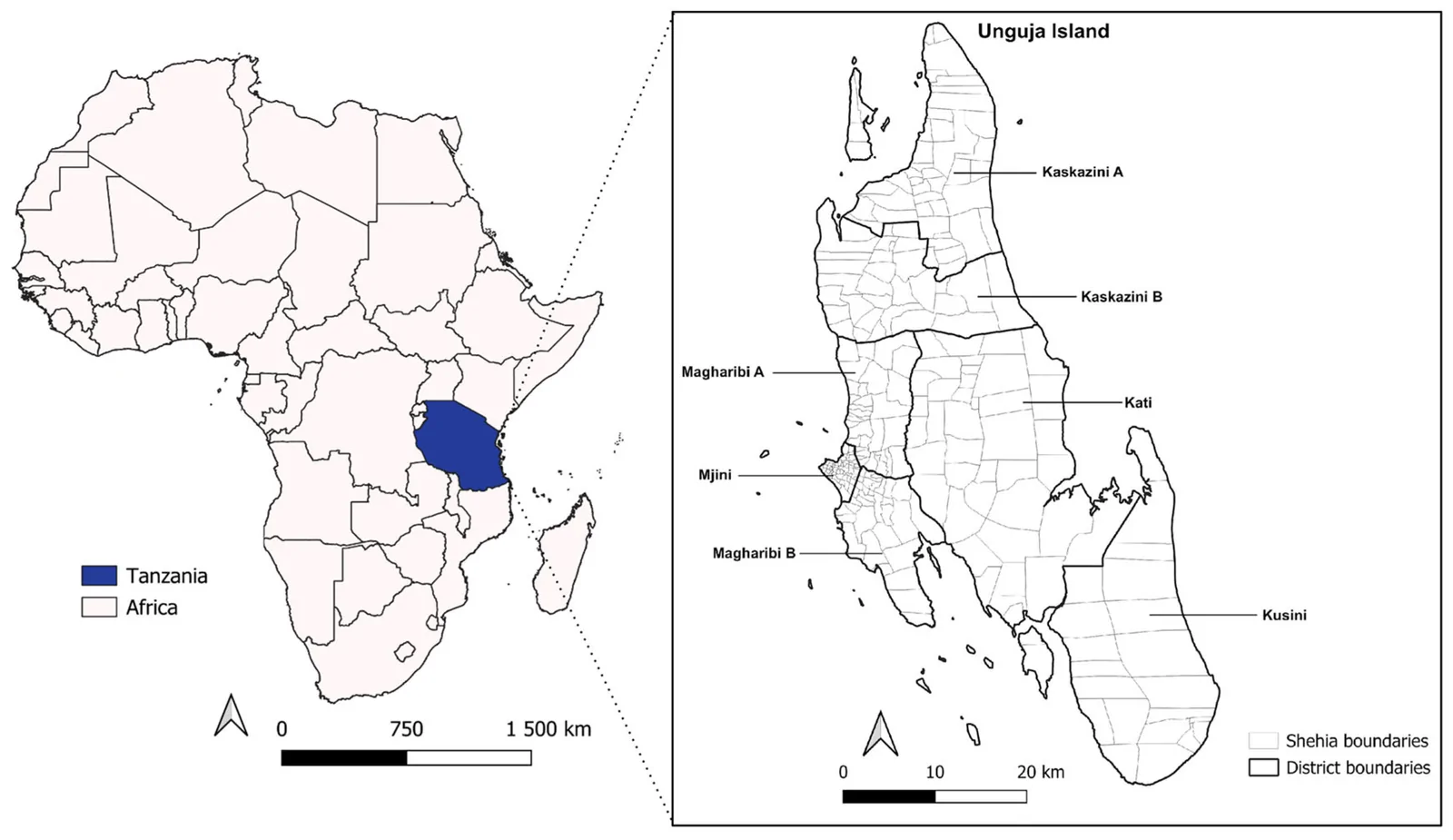
Map showing where Unguja Island is located and the seven districts found on the island. This image was created by the author using QGIS Desktop v3.44.2 [21].

Unguja Island is approximately 2460 square kilometers (km^2^) in size, and forms part of the Zanzibar Archipelago, which is situated 35 km (kilometers) off the east coast of The United Republic of Tanzania (URT). Unguja Island consists of three regions, which can be further divided into seven districts (Figure 1). Each district is further divided into 259 shehias (wards), which are the smallest administrative level on the island.

Mjini District, Magharibi A and Magharibi B, found on the western side of Unguja Island, are classified as urban and mixed districts due to their higher human population densities [22]. The other four districts are mainly classified as rural districts with lower human population densities (Kaskazini A, Kaskazini B, Kati, Kusini District).

### 2.2. Molecular Analysis of RABV from Used LFDs

#### 2.2.1. Used Lateral Flow Devices

The rabies-positive LFDs used in this study were collected from January 2022 to the end of 2024 as part of the RAIDER toolkit’s implementation to enhance active surveillance on Unguja Island (Table S1). These rapid tests were used according to the protocol provided in the RAIDER toolkit. This includes a brain sample collected using the straw sampling method through the occipital foramen [13]. The brain sample was then prepared for the LFD according to the manufacturer’s instructions (ADTEC Co., Tokyo, Japan).

All RABV-positive LFDs that had a positive direct fluorescent antibody test (DFAT) or DRIT brain sample were included in this study (*n =* 24). All metadata for the rapid tests were collected. The Rabies Ag Test (ADTEC Co., Japan) was used in the RAIDER protocol.

#### 2.2.2. Storage and Shipping of the LFDs

This study was approved by the University of Pretoria’s Faculty of Natural and Agricultural Science Research Ethics Committee and Animal Ethics Committee (Approval number NAS249/2024). The Department of Agriculture, Land Reform and Rural Development (DALRRD) approved the study under Section 20 of the Animals Disease Act, 1984 (Act number 35 of 1984) (Reference: 12/11/1/1/6823(HP)). Samples were imported into South Africa for analysis under veterinary import permit number 13/1/1/30/2/0-202504000192.

All LFDs (*n =* 24) were stored under refrigerated temperatures at the Department of Livestock Services (Ministry of Agriculture, Irrigation, Natural Resource and Livestock (MAINL)), situated in Maruhubi, Unguja Island (Zanzibar), and then shipped to the Transboundary Animal Diseases Programme (TAD-P), situated at the Agricultural Research Council—Onderstepoort Veterinary Research (ARC-OVR), for inactivation, whereafter samples were moved to the University of Pretoria, Lyssavirus Research Laboratory for further molecular analysis.

#### 2.2.3. Total RNA Extraction from the Used LFDs

The plastic cover of the LFD was removed using sterile scissors, and the whole nitrocellulose strip was detached from the LFD cassette. The entire nitrocellulose membrane was cut into pieces and placed in an Eppendorf tube containing 1 mL (milliliter) TriZol reagent (Invitrogen, Waltham, Massachusetts (MA), United States of America (USA)). Thereafter, all tubes were heat-inactivated at 65 °C for 30 min (minutes).

Total RNA extraction was performed from the RABV-positive LFDs (*n =* 24) using the Direct-zol™ RNA MiniPrep Plus kit (Zymo Research, Irvine, California (CA), USA) according to the manufacturer’s instructions, with the exception that the RNA was eluted in 2 steps as follows. In total, 50 μL DNase/RNase-Free water (H_2_O) was added directly to the column matrix and centrifuged for 30 s (seconds) to elute the RNA. An additional 50 μL DNase/RNase-Free H_2_O was added to the column matrix and centrifuged again to obtain a final volume of 100 μL eluted RNA.

#### 2.2.4. Reverse Transcription and Amplification of the Partial Nucleoprotein Gene Region

RABV RNA was reverse-transcribed using 17 μL RNA per reaction and 1 μL 001lys primer [23]. The RNA and 001lys primer were added to an Eppendorf tube and heated to 94 °C for 1 min, and then immediately placed on ice for 5 min. For each cDNA reaction, the following was added: 4.5 μL SuperScript reaction buffer (5×) (ThermoScientific, Waltham, MA, USA), 2.2 μL deoxynucleotide triphosphate (dNTP) mix (10 mM (millimolar)) (Promega), 0.4 μL SS Reverse transcriptase (40 U (enzyme units)) (ThermoScientific) and 0.4 μL of Ribolock^®^ Ribonuclease Inhibitor (40 U/μL) (ThermoScientific) per reaction. The Eppendorf tube was then incubated for 90 min at 42 °C. The enzyme was inactivated by incubating the tube at 70 °C for 15 min.

PCR amplification was performed using 10 μL of the generated cDNA along with the 001lys and 550B primers [23]. For each PCR reaction, the following reagents were added: 14 μL Nuclease-free H_2_O, 25 μL DreamTaq Master Mix (ThermoScientific), 0.5 μL 001lys (forward primer), and 0.65 μL 550B (reverse primer). The PCR cycle conditions are described by Malan et al. [24]. PCR amplification was repeated as above using 1 μL of the initial PCR product as a template. After the second round of amplification, the PCR products (*n* = 24) were electrophoresed on a 1% agarose gel and purified using the Zymoclean™ Gel DNA Recovery Kit from Zymo Research according to the manufacturer’s instructions.

#### 2.2.5. Sanger Sequencing

The 001lys/550B primers were used to generate the partial RABV N gene sequence. A mix of the following reagents was prepared in a DNA/RNA-free clean room in a 0.2 mL Eppendorf tube: 3 μL BigDye^®^ Terminator mix v3.1 (Applied Biosystems, Life Technologies, Waltham, MA, USA), 1 μL primer (10 pmol) and 7 μL purified PCR template. The Eppendorf tube was briefly centrifuged to collect all the reagents at the bottom of the tube. The tubes were placed in a thermocycler. The reaction conditions included denaturation at 94 °C for 60 s. There were 25 cycles of 94 °C performed for 10 s, 50 °C for 5 s, and 60 °C for 4 min. After amplification, the reactions were purified by ethanol precipitation. The sequencing reaction was transferred from the 0.2 mL Eppendorf tube to an appropriately labeled 0.6 mL Eppendorf tube. A volume of 1 μL EDTA (125 mM) was added to the reaction. A volume of 1 μL sodium acetate (NaOAc) (3 M (Molar)) was then added to the reaction. A volume of 25 μL 100% ethanol (EtOH) (molecular grade) was added to the reaction. The Eppendorf tube was vortexed and then incubated at room temperature for 15 min. The reaction was centrifuged at 10,000× *g* for 30 min. The supernatant was removed with a pipette and 300 μL of 70% EtOH was added to the tube. EtOH was sprayed on the edges of the tube and allowed to run down the inner walls. The reaction was then centrifuged at 10,000× *g* for 15 min. The supernatant was removed. This EtOH wash step was repeated, and the reaction was centrifuged at 10,000× *g* for an additional 15 min. The supernatant was removed, and the DNA pellet was allowed to air dry for 1 min at 94 °C.

The samples were submitted to the African Centre for Gene Technologies (ACGT) Sequencing facility at Forestry and Agricultural Biotechnology Institute (FABI) (situated at the University of Pretoria) for capillary electrophoresis using a ABI3500xL Genetic Analyzer (Applied Biosystems, ThermoFisher Scientific, Waltham, MA, USA).

The sequences generated from the Sanger sequencing of the forward and reverse reactions of the partial N gene were imported into CLC Main Workbench software (version 25.0.3) [25]. Each sequence chromatogram was visually inspected for quality (Phred score > 20) before further analysis. The forward and reverse sequences were assembled to generate a consensus sequence. The final consensus sequences were then trimmed to 405 nucleotides (nt) using CLC Main Workbench. The newly obtained RABV sequences (*n =* 24) were uploaded to the National Centre for Biotechnology Information (NCBI) GenBank to obtain unique accession numbers (PX458845 to PX458868).

#### 2.2.6. Phylogenetic Analysis of the Partial Nucleoprotein Gene Region

The phylogenetic analysis included the 24 RABV sequences isolated from the LFDs, as well as sequences from Unguja Island, mainland Tanzania, Botswana, Ethiopia, Kenya, Mozambique, South Africa, Zambia and Zimbabwe (Table S2).

MEGA11 was used to align a total of 86 RABV sequences using ClustalW [26] and trimmed to 405 nt. The best nucleotide substitution model for this dataset was determined to be GTR + G using the jModel Test 2 software (version 1.10) [27] based on the Akaike’s information criterion (AIC).

The phylogenetic analysis of the dataset was undertaken using the BEAST software package (version 10.5.0), which included the Broad-platform Evolutionary Analysis General Likelihood Evaluator (BEAGLE) library [28] using a Markov Chain Monte Carlo (MCMC) method for Bayesian inference [29]. The posterior distributions were analyzed using TRACER software (version 1.7.2) [30] to ensure adequate mixing and convergence before the Maximum clade credibility tree was summarized and visualized using FigTree software (version 1.4.4) [31].

### 2.3. Analysis of Rabies Surveillance in Unguja Island (2016 to 2024)

#### 2.3.1. Data Collection and Curation

Data from RABV samples obtained as part of the routine rabies surveillance carried out by the Government of Zanzibar from July 2016 to December 2024 were included in this study. Brain tissue samples were collected from suspected rabid animals, as part of the routine rabies surveillance program implemented by the Department of Livestock Service (Ministry of Agriculture, Irrigation, Natural Resource and Livestock (MAINL)) in collaboration with GARC. Rabies is a notifiable disease in Zanzibar, and all animal health professionals are thus mandated to undertake routine animal rabies surveillance throughout the country. As such, animal health professionals are authorized by law to humanely euthanize and collect samples from any suspected animals for rabies diagnosis through Zanzibar’s Animal Resource Management Act, Number 11 of 1999.

The rabies surveillance data collected between July 2016 and December 2024 (Table S3) were combined and consolidated into a single Excel spreadsheet [32]. The University of Pretoria’s Faculty of Natural and Agricultural Sciences Ethics Committee approved the use of the raw surveillance data for retrospective analysis (Approval Number NAS122/25).

For this study, the surveillance data consisted of (i) the date of diagnosis (day-month-year), (ii) species subjected to diagnosis, (iii) location of sampling (Shehia and Global Positioning System (GPS) coordinates), and (iv) diagnostic outcome (positive or negative), and these were used for subsequent analyses. The species in the dataset were categorized as follows: domestic animals (all domestic dog and domestic cat species), livestock (bovine, caprine, equine and ovine species), and wildlife (all wildlife species, including bat species and rodents).

The geographic distribution for all positive and negative rabies cases throughout Unguja Island was visualized using the QGIS (Quantum Geographic Information System) Desktop v3.44.2 [21], which relies on the use of GPS coordinates associated with each sample. In addition, the most recent shapefile for Unguja Island was used in QGIS Desktop to visualize all the maps generated in this study [33].

#### 2.3.2. Spatio-Temporal Analysis of Rabies Cases in Unguja Island

Individual datasets were created for all animals, domestic animals, livestock and wildlife. Each dataset consisted of the following information in order: date (year-month-day/ YYYY-MM-DD) (ISO 8601 standard [34]), name of shehia, number of positive cases, number of negative cases, and exact latitude and longitude coordinates in decimal degree (DD) format.

Each dataset was imported into the SaTScan™ software version 10.3.2 [35] for space–time analysis from 2016 to 2024. The Bernoulli space–time probability model was used to determine statistically significant disease hotspot clusters based on the number of positive cases (presence of rabies) and negative cases (absence of rabies) in a geographical region. The population at risk parameter was set to 0.25. The Monte Carlo Replications were set at 99,999, which assesses the statistical significance of the clusters. Statistically significant disease clusters were defined as having a *p*-value below 0.05 (*p* < 0.05). Only statistically significant clusters were included. High-rate clusters were defined as areas that had more observed rabies cases than expected under the null hypothesis, whereas low-rate clusters were defined as areas that had fewer observed rabies cases than expected. The relative risk (RR) was also calculated for each significant cluster, and the time period for which the cluster was present was also determined. All maps with SaTScan results were created using QGIS Desktop (version 3.44.2).

#### 2.3.3. Calculating Testing Rates for Rabies Surveillance Data on Unguja Island

Testing rates were calculated using the method described by Minhaj et al. [36] and the Tanzania 2022 human census data [22]. The rabies surveillance data from 2016 to 2024 were also used for these calculations.

The all-animal-per-human testing rate (AAHR) (Equation (1)) and domestic-animal-per-human testing rate (DAHR) (Equation (2)) were calculated for each shehia on Unguja Island using the formulae below.

(1) $$AAHR\;=\;\frac{Average\;number\;of\;all\;animals\;tested/year}{Estimated\;human\;population}\times 100,000$$

(2) $$DAHR\;=\;\frac{Average\;number\;of\;domestic\;animals\;tested/year}{Estimated\;human\;population}\times 100,000$$

Minhaj et al. [36] has defined threshold values for these testing rates to determine if the surveillance data are adequate or not (AAHR 1.9 and DAHR 0.8). These thresholds were determined by analyzing publicly available rabies surveillance data from countries that have a functioning passive rabies surveillance system and are endemic to canine rabies. The testing rates were calculated by descriptive analysis, for which the AAHR and DAHR thresholds were defined by using the first quartile (Q1) as the conservative cut-off value for adequate surveillance. Shehias that have a testing rate above the threshold have adequate surveillance and shehias below the threshold have poor surveillance and rabies testing. These testing rates for each shehia were visualized using QGIS Desktop.

#### 2.3.4. Calculation of Dog Density Estimates

Dog density estimates were calculated, since dogs are the primary rabies reservoir and their population density influences the transmission risk to humans and other species. Therefore, this can be used to determine whether regions with poor surveillance have dog densities that would allow rabies transmission.

The GARC technical report [37,38] from 2022 combined previous dog population estimates from WAP, along with multi-year vaccination numbers from Unguja Island to calculate the estimated number of dogs per shehia. These estimates represent the total dog population. From this, using the area (km^2^) of the shehia [39] and dividing it with the dogs per shehia, the dog density per shehia was calculated (Equation (3)).

(3) $$Dog\;density\;=\;\frac{Dog\;population}{Area\;\left({km}^{2}\right)}$$

Some shehias had no data on the estimated dog population, and therefore the dog density could not be calculated. Thus, using the dog population for each shehia with available data and dividing it with the human population for that shehia, a Human-to-Dog Ratio (HDR) was calculated (Equation (4)) for each shehia that had available data. Using information from CityPopulation et al. [39], all the shehias was classified as “urban”, “mixed” or “rural”. Along with the area (km^2^) of the shehia, this allowed estimated human:dog ratios to be determined for “urban”, “mixed” or “rural” shehias.

(4) $$Human\;-\;to\;-\;Dog\;Ratio\;=\;\frac{Human\;population}{Dog\;population}$$

The average HDR for all urban and rural areas was then calculated (Table 1). For mixed shehias, the average of the estimated urban and rural HDR (356.13 and 199.60) were used to calculate the HDR as 277.86 (Table 1).

Using these estimated HDRs and the 2022 human census data, the dog density per shehia (km^2^) was calculated for the unknown shehias. This was done by dividing the human population per shehia by the HDR to get the dog population per shehia (Equation (4)). The dog population per shehia was then divided by the area (km^2^) to obtain the dog density (Equation (3)).

Hampson et al. [40] showed that a dog density as low as 1.36 dogs per km^2^ in a geographical region could maintain rabies transmission in the dog population. Therefore, as previously done by Malan et al. [7], the threshold value of 1.36 dogs per km^2^ was also used to determine in which shehias rabies transmission can theoretically be maintained in the dog population. The estimated dog densities for each shehia were also visualized using QGIS Desktop.

## 3. Results

### 3.1. Recent Molecular Epidemiology of Rabies on Unguja Island (2022–2024)

#### 3.1.1. Sample Set of RABV Included in the Molecular Epidemiological Analysis of the Partial Nucleoprotein Gene

The RABV sequences included in this molecular epidemiological analysis consisted of 24 partial N gene sequences generated in this study (Table S1) and previously published sequences from Zanzibar archipelago and mainland Tanzania, Kenya, Ethiopia and southern Africa (South Africa, Botswana, Mozambique, Zambia and Zimbabwe) (*n =* 62) (Table S2). For these previously published sequences, the species from which RABV was isolated included African civet (*Civettictis civetta*) (*n =* 2), bovine (*n =* 4), canine (*n =* 52), caprine (*n =* 7), donkey (*Equus asinus*) (*n =* 1), equine (*n =* 1), feline (*n =* 10), jackal (unknown species) (*n =* 2), human (*n =* 1), primate (unknown species) (*n =* 1), vervet monkey (*Chlorocebus pygerythrus*) (*n =* 1), wild cat (unknown species) (*n =* 1) and unknown species (*n =* 3).

#### 3.1.2. Molecular Epidemiological Phylogenetic Analysis of the Partial N Gene Sequences of the RABVs from Unguja Island, Tanzania and Other African Countries

**Figure 2:**
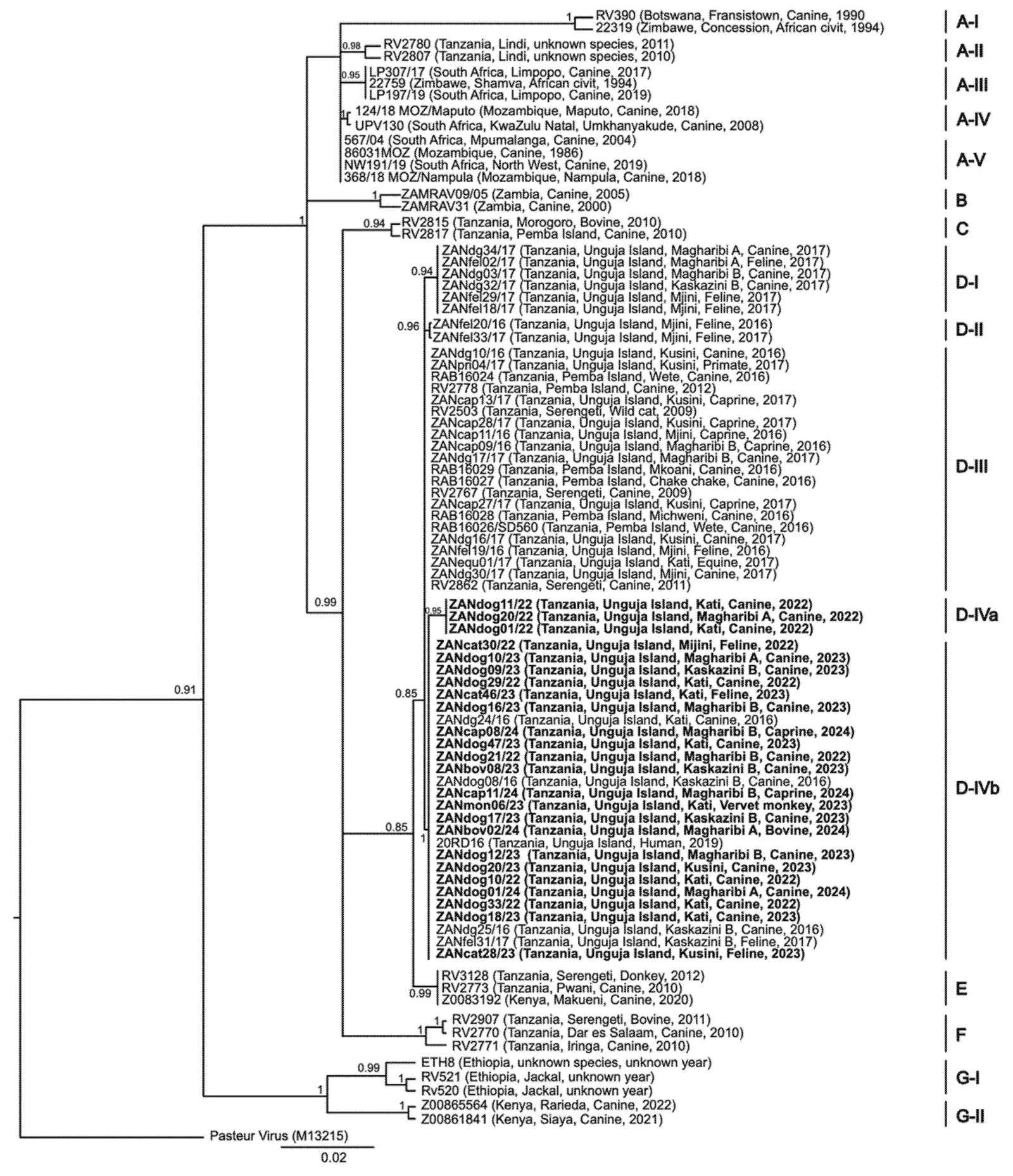
Maximum clade credibility phylogenetic tree of the partial N gene for RABVs that originated from various regions in the Zanzibar Archipelago, mainland Tanzania, Kenya, Ethiopia, Mozambique, South Africa, Botswana, Zambia, and Zimbabwe (Tables S1 and S2 [24,41,42,43,44,45,46,47,48,49,50,51,52]). Sequences generated in this study are indicated in bold on the phylogenetic tree. All branches that had a posterior probability below 0.75 were collapsed. The branch lengths are proportional to the homology between the sequences. The scale bar represents 2 nucleotide substitutions/changes per 100 positions. The Pasteur virus was used to root the phylogenetic tree. Clades A to F belong to the Africa 1b lineage [53], while clade G belongs to the Africa 1a lineage [53,54].

**Figure 3:**
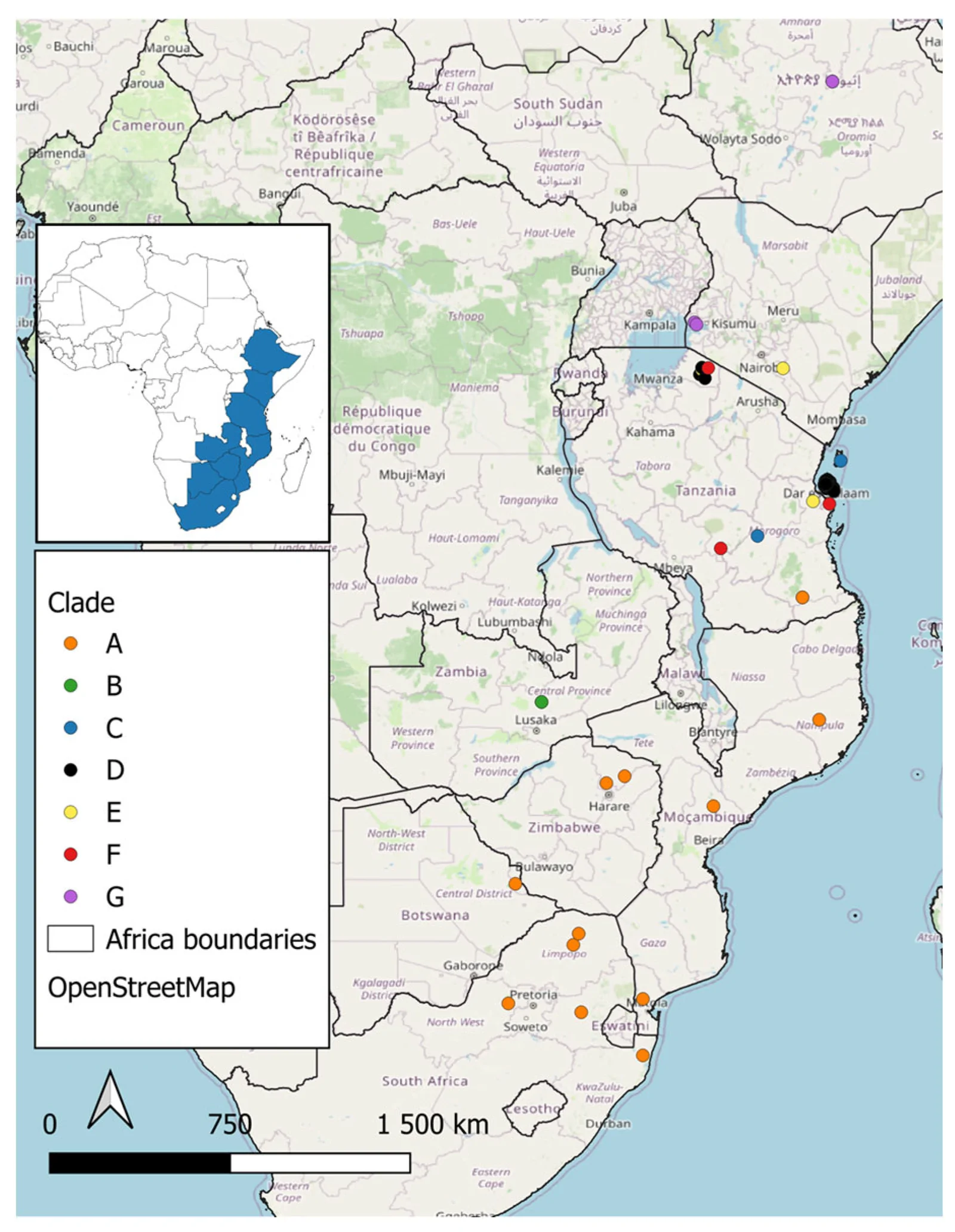
Map of eastern and southern Africa showing the geographical distribution of the RABV isolated for each clade (clade A–G) from the phylogenetic analysis of the partial N gene in this study. Location data for clade B (Zambia), as well as from Botswana, Mozambique, Zimbabwe, and Ethiopia, are not available and are represented using a centroid coordinate. This image was created by the author using QGIS Desktop version 3.44.2.

The partial N gene sequences included in this study originated from southern and eastern Africa and were separated into seven clades (Clades A–G) (Figure 2 and Figure 3) supported with high posterior probability (>0.85). Clade A RABV sequences originated from southern Africa, which included Botswana, Mozambique, SA, Tanzania and Zimbabwe. Clade B RABVs originated from Zambia. Clade C consisted of RABVs from Morogoro district (one bovine) on mainland Tanzania and Pemba Island (one canine). Clade D isolates were from Unguja Island, Pemba Island and Mainland Tanzania (Serengeti district). Clade E RABV sequences originated from Kenya and mainland Tanzania (Serengeti and Pwani district) and were isolated from a donkey (*n =* 1) and canines (*n =* 2). Clade F sequences were only from mainland Tanzania (Serengeti, Dar es Salaam and Iringa district) and isolated from canines (*n =* 3). Clade G consists of RABV sequences from Kenya and Ethiopia.

**Figure 4:**
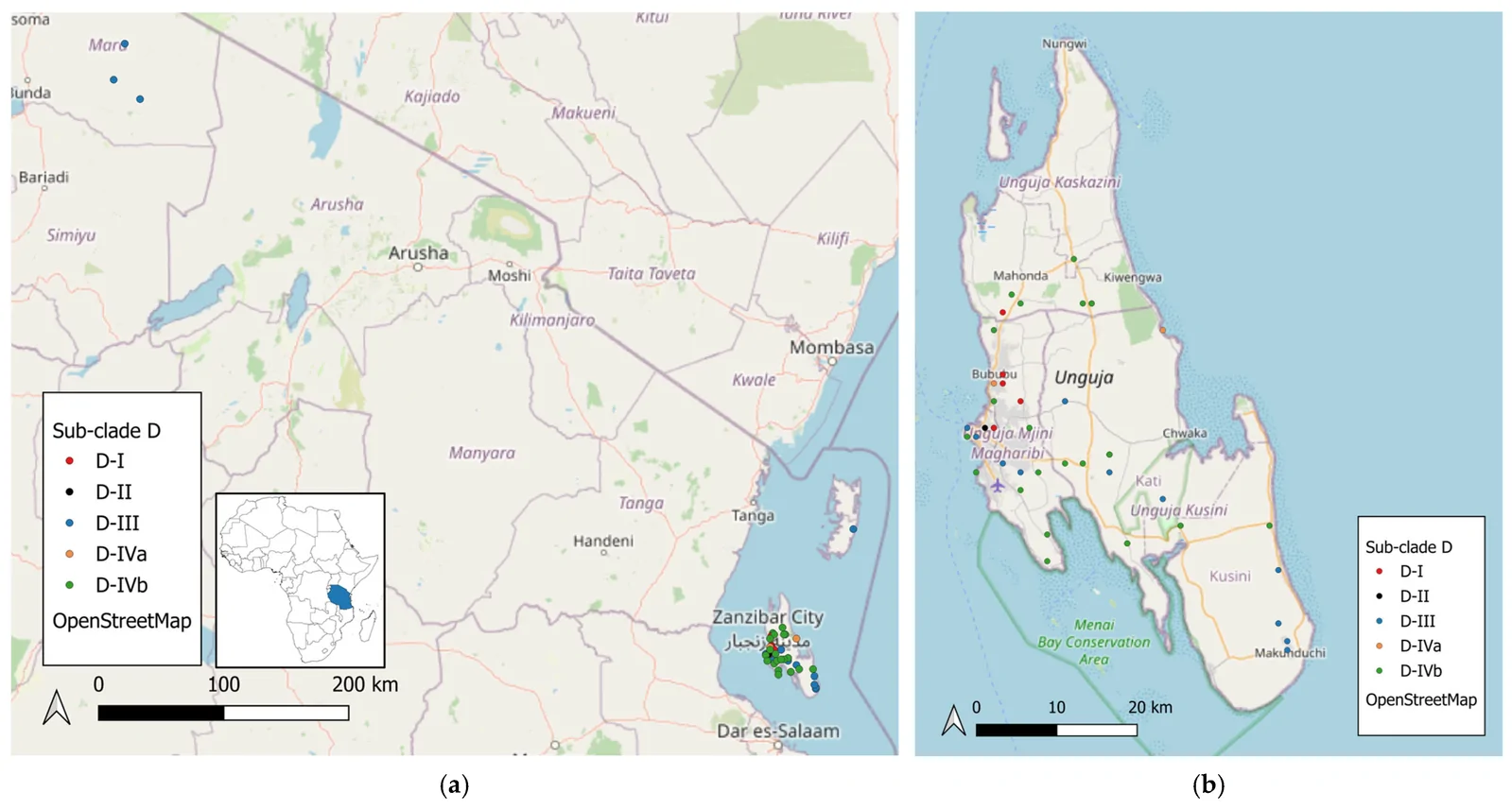
(**a**) Map of eastern Tanzania and the Zanzibar Archipelago that shows the geographical distribution of the RABV isolated sub-clade D from the phylogenetic analysis of the partial N gene in this study. (**b**) Map showing the geographical distribution of sub-clade D RABVs on Unguja Island. This image was created by the author using QGIS Desktop version 3.44.2.

Specifically focusing on where the newly generated RABV sequences were grouped, clade D (Figure 2, Figure 3 and Figure 4a,b) consisted of 58 RABVs divided into four sub-clades (D-I to D-IVb), which represent RABV samples from Unguja Island, Pemba Island and mainland Tanzania (Serengeti district). Clade D-I consisted of six isolates that were collected from canines (*n* = 3) and felines (*n* = 3). This clade is restricted to the western regions of Unguja Island during 2017.

Clade D-II consisted of two RABVs that were collected from felines (*n* = 2) over a period of two years (2016 to 2017). This clade is restricted to the urban central regions of Unguja Island (Mjini district).

Clade D-III consisted of 21 RABVs that were collected from canines (*n* = 12), caprine (*n* = 5), equine (*n* = 1), primate (*n* = 1) and wild cat (*n* = 1). The isolates from this clade originated from the Serengeti district in mainland Tanzania, Pemba Island and Unguja Island.

Clade D-IV consisted of 29 RABVs that originated geographically only from Unguja Island. Clade D-IV can further be phylogenetically divided into clade D-IVa and clade D-IVb (Figure 4b). Clade D-IVa isolates were collected only from canines (*n* = 3) during 2022. Clade D-IVb were collected from bovine (*n* = 2), canine (*n* = 16), caprine (*n* = 2), feline (*n* = 4), human (*n* = 1) and vervet monkey (*n* = 1) from 2016 to 2024. These sub-clades were not geographically separated. Clade D-IVa is distributed over the island, while clade D-IVb is located in the central regions of Unguja Island.

### 3.2. Analysis of Rabies Surveillance on Unguja Island (2016–2024)

#### 3.2.1. Overview of All Samples Submitted for Rabies Testing in Unguja Island, 2016–2024

A total of 165 samples were submitted for rabies testing from July 2016 to the end of 2024, with 53% (87/165) being rabies-negative and 47% (78/165) being rabies-positive (Table 2). Most of the positive cases originated from domestic animals (67%), followed by livestock (24%) and then wildlife (9%) (Table 2).

**Figure 5:**
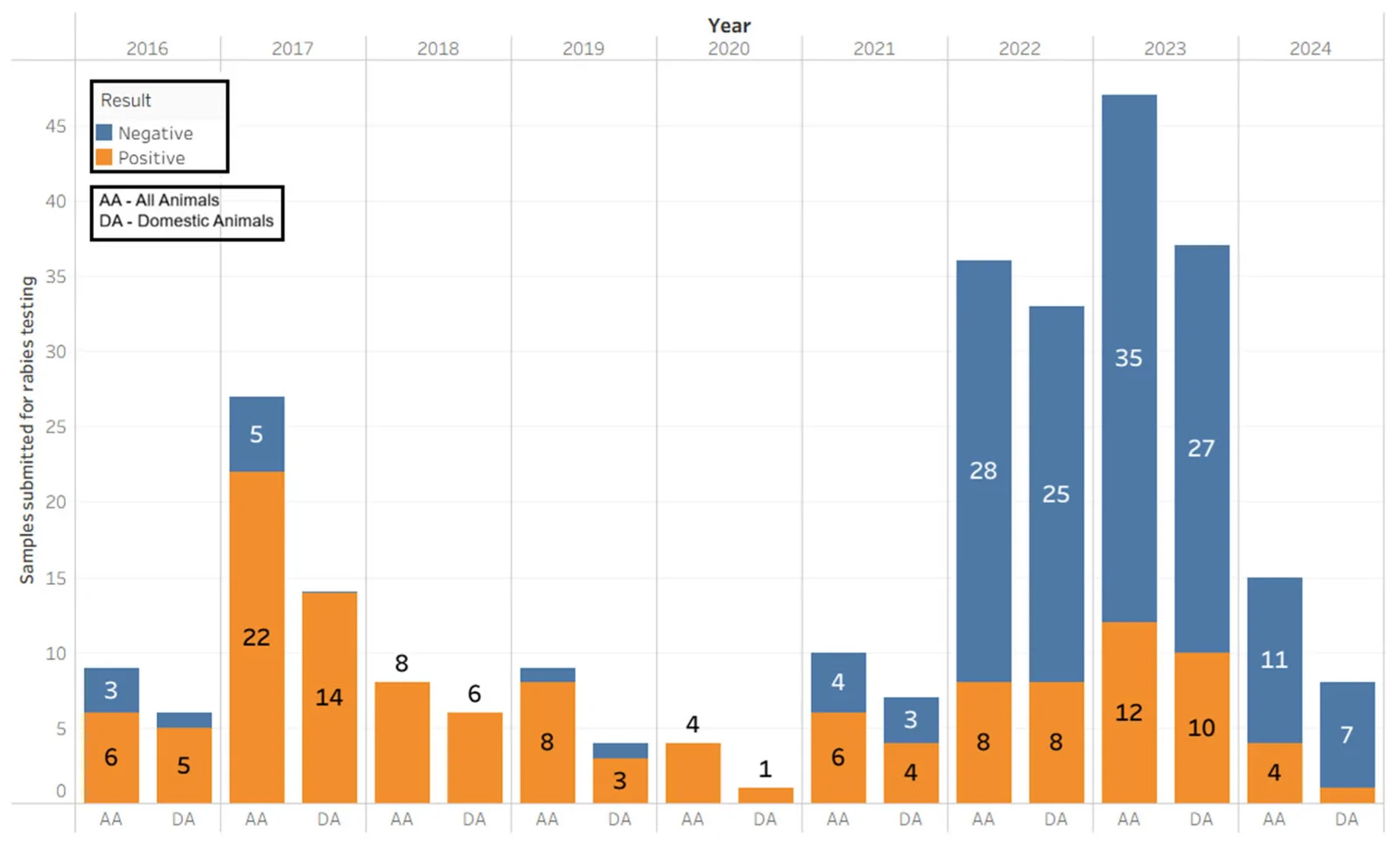
Suspected rabies samples submitted per year and test results for all animals and domestic animals. This image was created by the author using Tableau Desktop 2023.1.2 [55].

**Figure 6:**
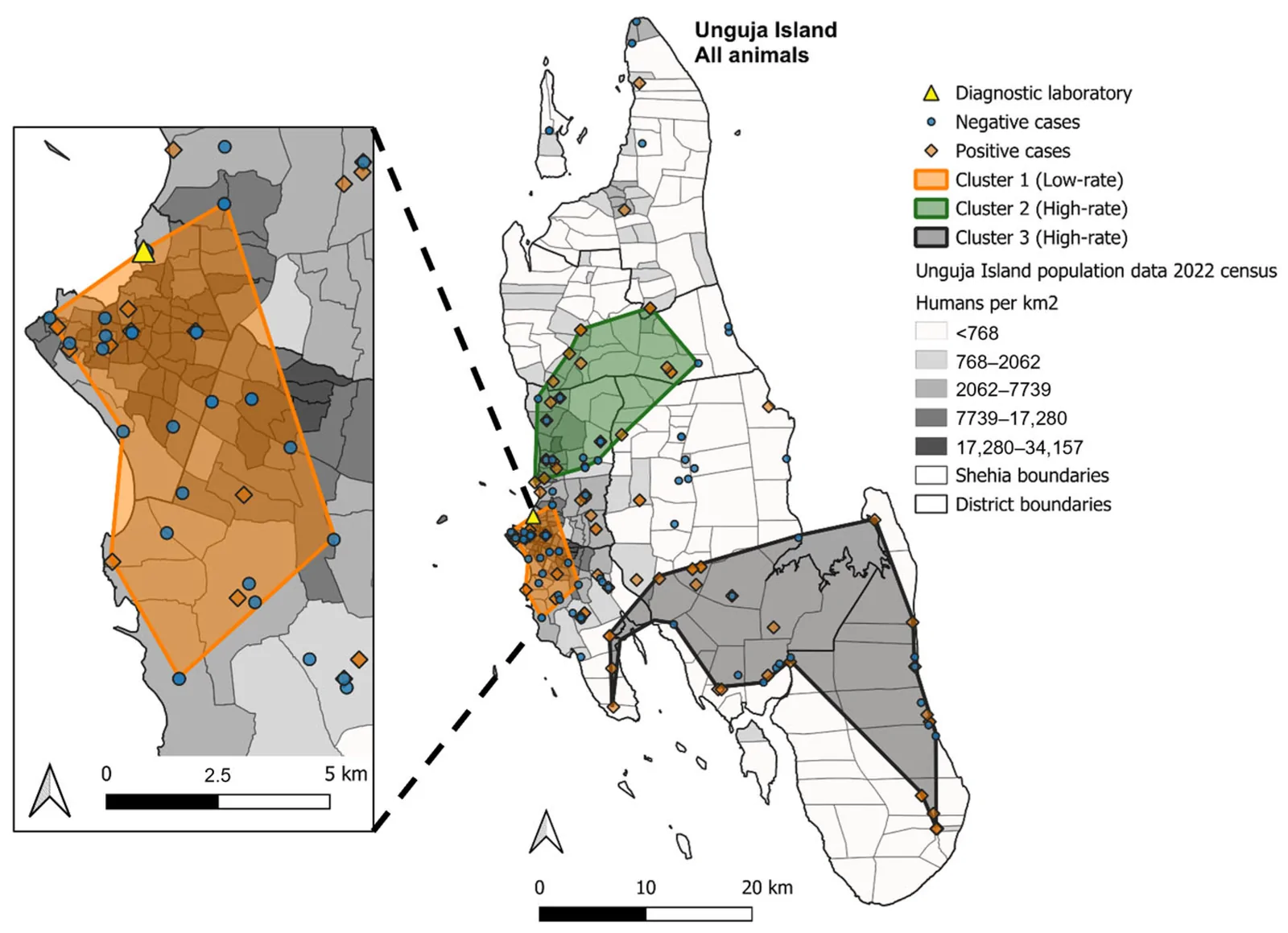
Geographic distribution of the positive and negative rabies cases, significant disease clusters in all animals in Unguja Island between 2016 and 2024, and the human population density. This image was created by the author using QGIS Desktop v3.44.2.

From Figure 5, it can be seen how many samples were submitted for rabies testing per year, along with the test result. From the start of the study period until 2021, there were few submissions, but almost all submissions were rabies-positive, except for 2017, which had 27 submissions that year (22/27 were positive). Following on from the implementation of the RAIDER toolkit from 2022 onwards, there was a gradual increase in submissions as well as an increase in samples that tested negative. Sample submissions decreased again in 2024. When looking at the geographic distribution of all the cases submitted from 2016 to 2024 (Figure 6), there is a widespread distribution over the island. The majority of samples were submitted from the western urban part of the island (Mjini, Magharibi A and Magharibi B).

From the space–time analysis of the surveillance data for all animal species in Unguja Island using SaTScan™, three significant disease clusters were detected (Table 3, Figure 6). Cluster 1 is a low-rate cluster and was located in the urban and mixed areas of the island, covering almost all of Mjini, the southern part of Magharibi A and the northwestern part of Magharibi B. Cluster 2 and cluster 3 are both high-rate clusters. Cluster 2 was located over most of Kaskazini B and small parts of Magharibi A, Kaskazini A and Kati. Cluster 3 was located over large parts of Kati and Kusini and a small region in Magharibi B. In Table 3, cluster 1 is the most recent, being detected from November 2020 to November 2024, whereas cluster 2 and cluster 3 were observed from July 2016 to November 2020. Cluster 1 had a lower number of observed cases than expected, which is consistent with the low RR, whereas cluster 2 and cluster 3 had more observed cases than expected (17 and 15 cases, respectively), indicated by the high RR (Table 3).

Figure 6 shows the significant disease clusters with the geographic distribution of the rabies-positive and -negative cases included on the map. Cluster 1, which is located in the western part of the island, shows 32 samples (represented by blue dots and orange diamonds) that were submitted for rabies testing, visually looking like a hotspot for rabies transmission, but representing a low-rate cluster. Clusters 2 shows fewer submitted samples (26 samples) than cluster 1. Cluster 3 shows more samples (34 samples) that were submitted over a larger geographic area than cluster 1. Visually, clusters 2 and 3 do not look like hotspots, but SaTScan™ detected these areas as high-risk clusters for rabies transmission. This shows the value of space–time analysis when compared with only the sample distribution on a map.

**Figure 7:**
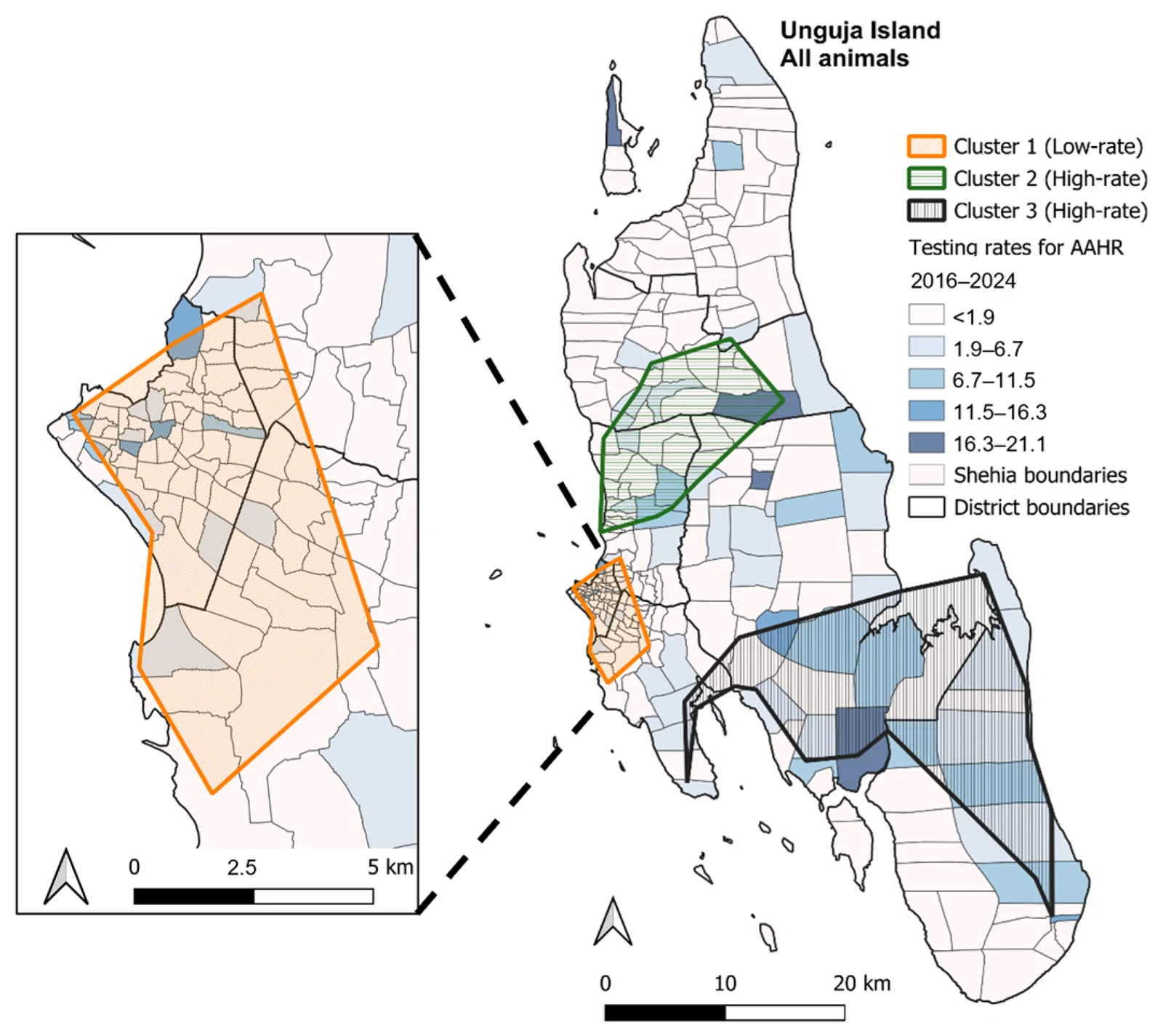
Significant disease clusters and AAHR for all animals in Unguja Island (2016–2024). This image was created by the author using QGIS Desktop v3.44.2.

Testing rates were calculated to determine whether the absence of a disease cluster is due to the true absence of disease or due to poor surveillance. The AAHR was calculated and visualized for all animal species from 2016 to 2024 (Figure 7). Out of the 259 shehias, only 62 shehias (24%) had an AAHR above the threshold (>1.9). The shehia with the highest testing rate was Pete (21.08), followed by Uzini (20.42), both located in Kati, followed by Mtakuja (20.27) located on Tumbatu Island (part of Kaskazini A), followed by Kilombero (20.21) located in Kaskazini B. Large parts of Kaskazini A and Kaskazini B, as well as most parts in Mjini, had testing rates below the required threshold (AAHR < 1.9). The other shehias with testing rates equal to or higher than the threshold (AAHR: 1.9–16.3) are scattered across the island.

When comparing the statistically significant disease clusters with the testing rates (Figure 7), clusters 1 and 2 span over large areas where the testing rates are below the required threshold, although there are some shehias with high testing rates. In cluster 1, 82% (70/85) of the shehias had below-threshold testing rates (AAHR < 1.9), and in cluster 2, 69% (25/36) of shehias had below-threshold testing rates. For cluster 3, the majority of the area (63–19/30 shehias) had testing rates above the threshold. For all three clusters, most of the surrounding shehias also fell below the AAHR threshold.

#### 3.2.2. RAIDER and AAHR

**Figure 8:**
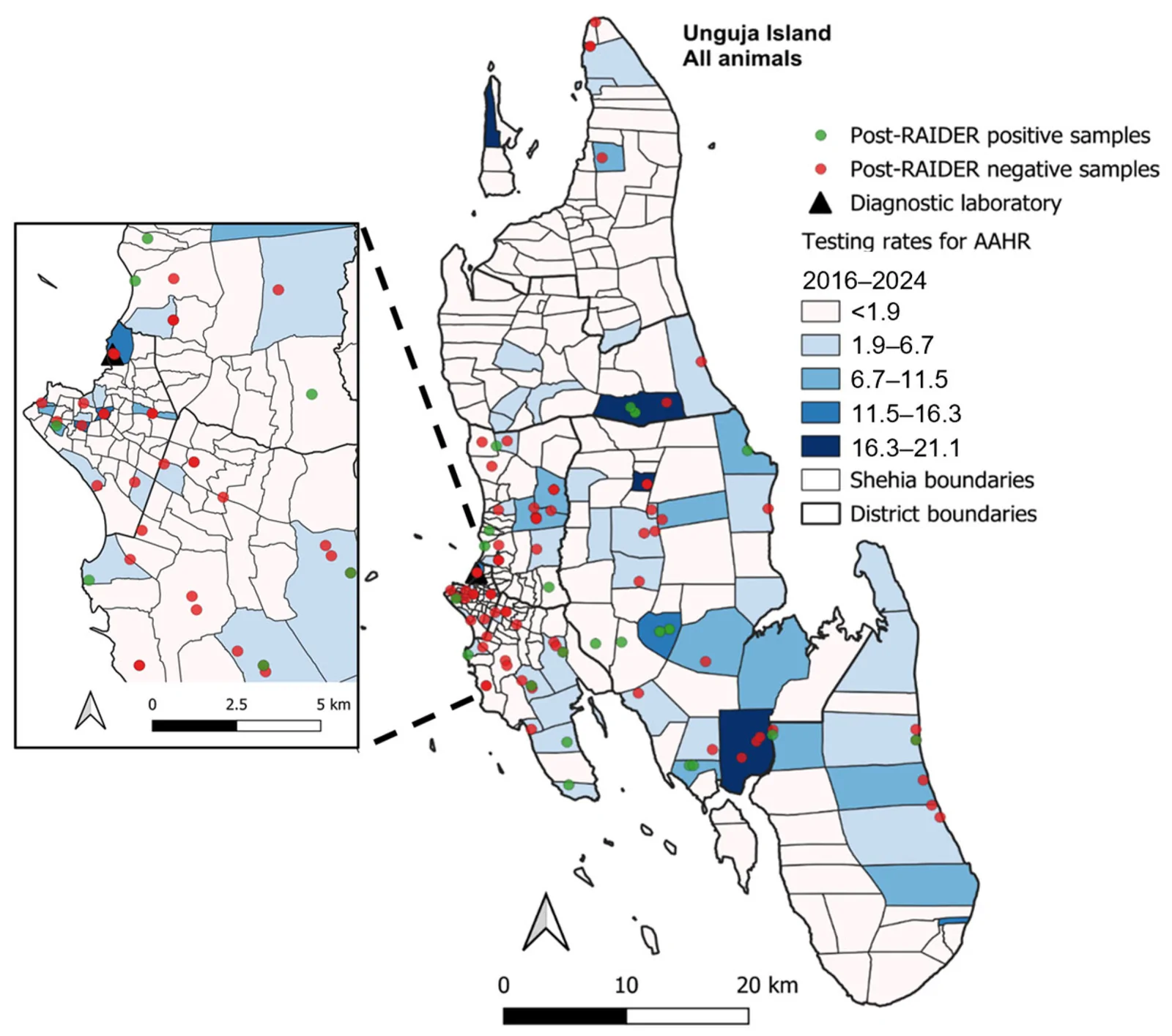
Geographic distribution of rabies-positive and rabies-negative samples submitted after the implementation of the RAIDER toolkit (2016–2024) and AAHR in Unguja Island. This image was created by the author using QGIS Desktop v3.44.2.

Figure 8 shows the geographic distribution of the rabies-positive and -negative cases after the implementation of the RAIDER toolkit together with the AAHR. Most of the submitted samples were from shehias that had testing rates equal to or higher than the threshold values (AAHR > 6.7). Most of the rabies-negative cases were located in the western areas of the island (Mjini, Magharibi A and B), whereas rabies-positive cases were scattered over the island, except in the northern districts. A few shehias, where no samples were submitted after the implementation of RAIDER, also had testing rates above the threshold.

#### 3.2.3. Domestic Animals Submitted for Rabies Testing in Unguja Island, 2016–2024

A total of 116 samples from domestic animals were submitted between 2016 and 2024, with 55% (64/116) being negative (Table 4). Domestic dogs accounted for 71% of total submissions, of which 79% (41/52) of all rabies-positive cases were from domestic dogs. Domestic cats accounted for 29% of total submissions, and 21% of all rabies-positive cases were from cats.

**Figure 9:**
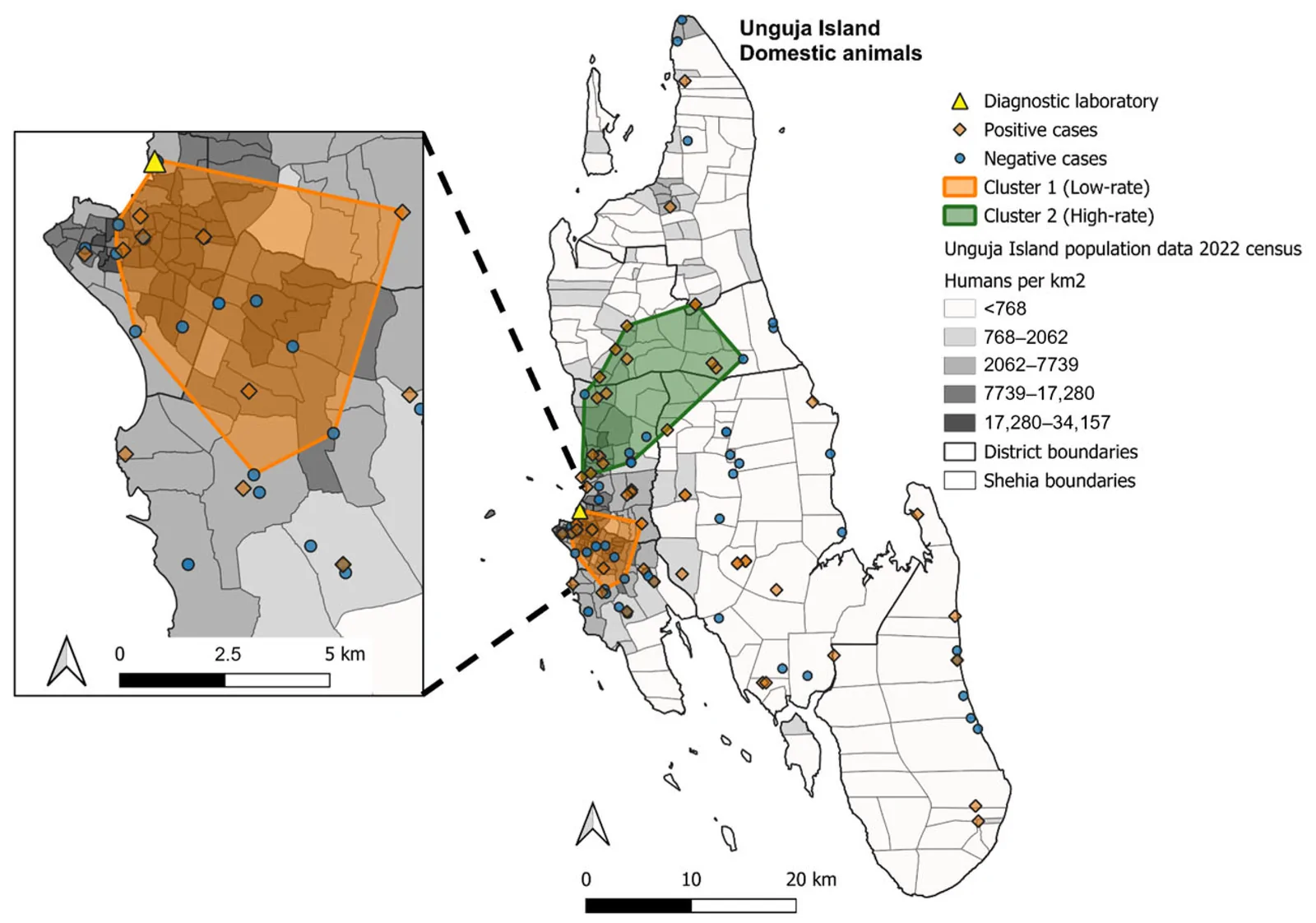
Geographic distribution of the rabies-positive and) rabies-negative cases, significant disease clusters in domestic animals in Unguja Island between 2016 and 2024 and the human population density. This image was created by the author using QGIS Desktop v3.44.2.

During 2016 to 2021, sample submission was low, with the exception of 2017 (Figure 5). A gradual increase in submissions is seen as well, as a big increase in the number of negative test outcomes, from 2022 onwards, with a decrease in sample submissions in 2024. From the geographical distribution of the cases submitted for domestic animals (Figure 9), it can be seen that most of the positive and negative cases were concentrated in the urban (Mjini) and mixed areas (Magharibi A and B).

From the space–time analysis of the surveillance data for domestic animals in Unguja Island using SaTScan™, two significant disease clusters were detected (Table 5, Figure 9). Cluster 1 is a low-rate cluster and was located in the urban and mixed areas of the island, covering almost all of Mjini, the southern part of Magharibi A and the northwestern part of Magharibi B. Cluster 2, a high-rate cluster, was located over most of Kaskazini B and small parts of Magharibi A, Kaskazini A and Kati. When looking at Table 5, cluster 1 is the most recent, being detected from November 2020 to November 2024, whereas cluster 2 was observed from July 2016 to November 2020. Cluster 1 had zero observed positive cases, despite having an expected 7.6 cases, which is consistent with the low RR (RR = 0), whereas cluster 2 contained more observed cases than expected (11 cases), consistent with the high RR (Table 5).

Figure 9 shows the significant disease clusters that were detected from the space–time analysis with the geographic distribution of the rabies-positive and -negative cases. Cluster 1, located in the western part of the island, shows 20 samples that were submitted for rabies testing in a small geographic area, visually looking like a hotspot for rabies transmission, but representing a low-rate cluster. Cluster 2 shows fewer samples (22 samples) that were submitted from a larger geographic area. As with cluster 2 from All Animals, this area does not look like a hotspot, but SaTScan™ detected this area as a high-risk cluster for rabies transmission, again demonstrating the value of space–time analysis, rather than only relying on the sample distribution on a map.

**Figure 10:**
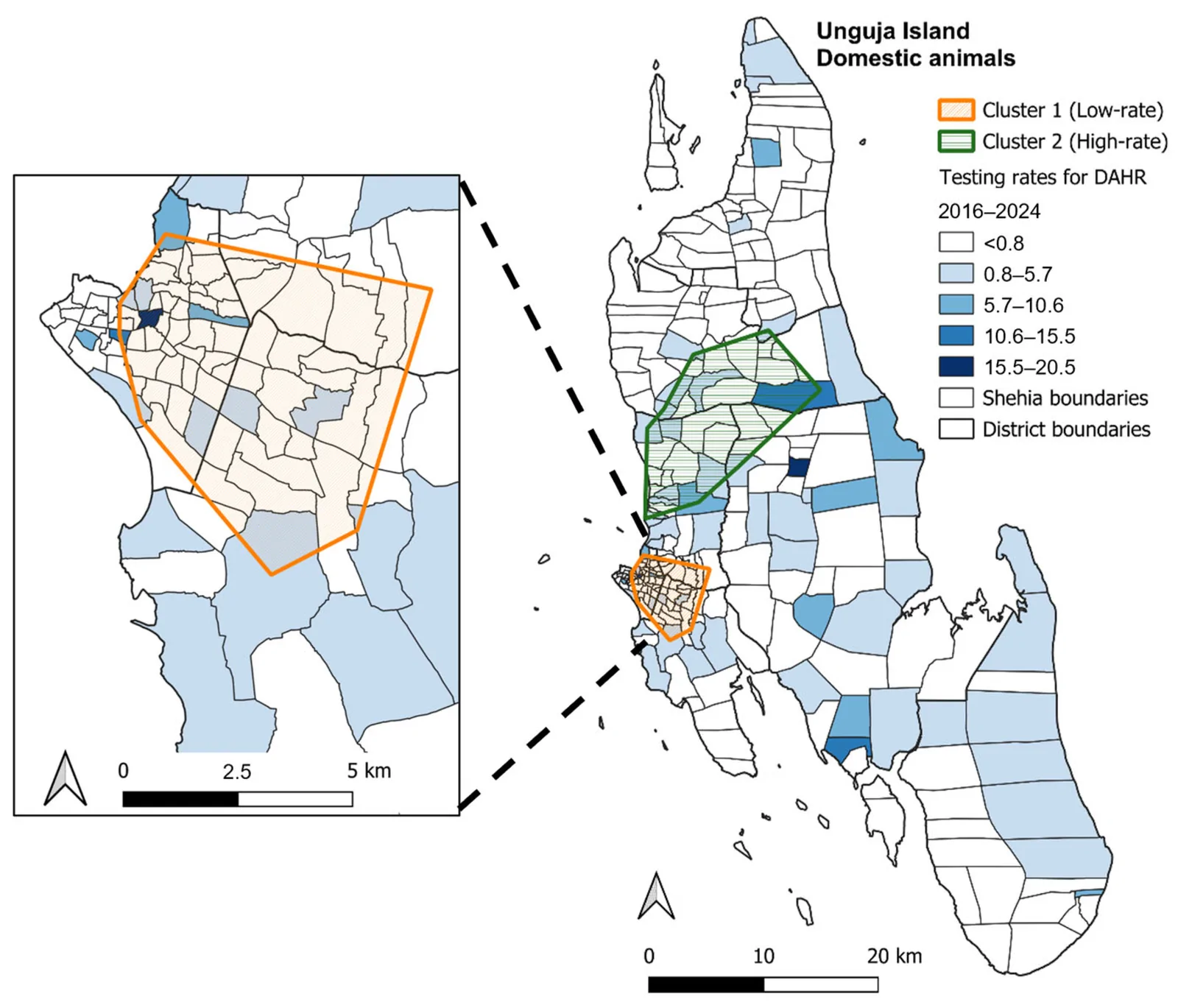
Significant disease clusters and DAHR testing rates for domestic animals in Unguja Island (2016–2024). This image was created by the author using QGIS Desktop v3.44.2.

Testing rates were calculated to determine if the presence or absence of a disease cluster is due to the true prevalence of the disease, or due to poor surveillance. The DAHR was calculated and visualized for all domestic animals for 2016 to 2024 (Figure 10). Out of the 259 shehias, only 64 shehias (25%) had a DAHR testing rate above the threshold (0.8). The shehia with the highest testing rate was Uzini (20.42) located in Kati, followed by Mikunguni (20.27) located in Mjini. Large parts of Kaskazini A, some parts of Kaskazini B as well as most parts in Mjini had testing rates below the required threshold. The other shehias with above-threshold testing rates (0.8–10.6) are scattered across the island.

When comparing the statistically significant disease clusters with the DAHR (Figure 10), clusters 1 and 2 span over large areas where the testing rates are below the required threshold and have neighboring shehias that are also below the threshold. Cluster 1 has 83% (60/72) shehias with below-threshold testing rates, and cluster 2 has 57% (20/35) shehias with no testing rates.

#### 3.2.4. Dog Density

Estimated dog population numbers [33] were used to calculate approximate dog densities for each shehia. Unfortunately, most areas in Mjini and some areas in other districts had no data to determine the dog densities (97 unknown shehias).

Based on the estimated HDR ratios calculated in Table 1, the ratios obtained were higher than expected. The HDR ranged from 199.60 for rural to 356.13 for urban, with mixed regions as the average (277.86). As expected, the average HDR for rural shehias are lower than urban shehias, which indicates more dogs relative to humans in rural settings. Based on the HDR for each shehia, the dog population was estimated per shehia, and the total estimated dog population was found to be 9865 dogs for Unguja Island (Table S3).

**Figure 11:**
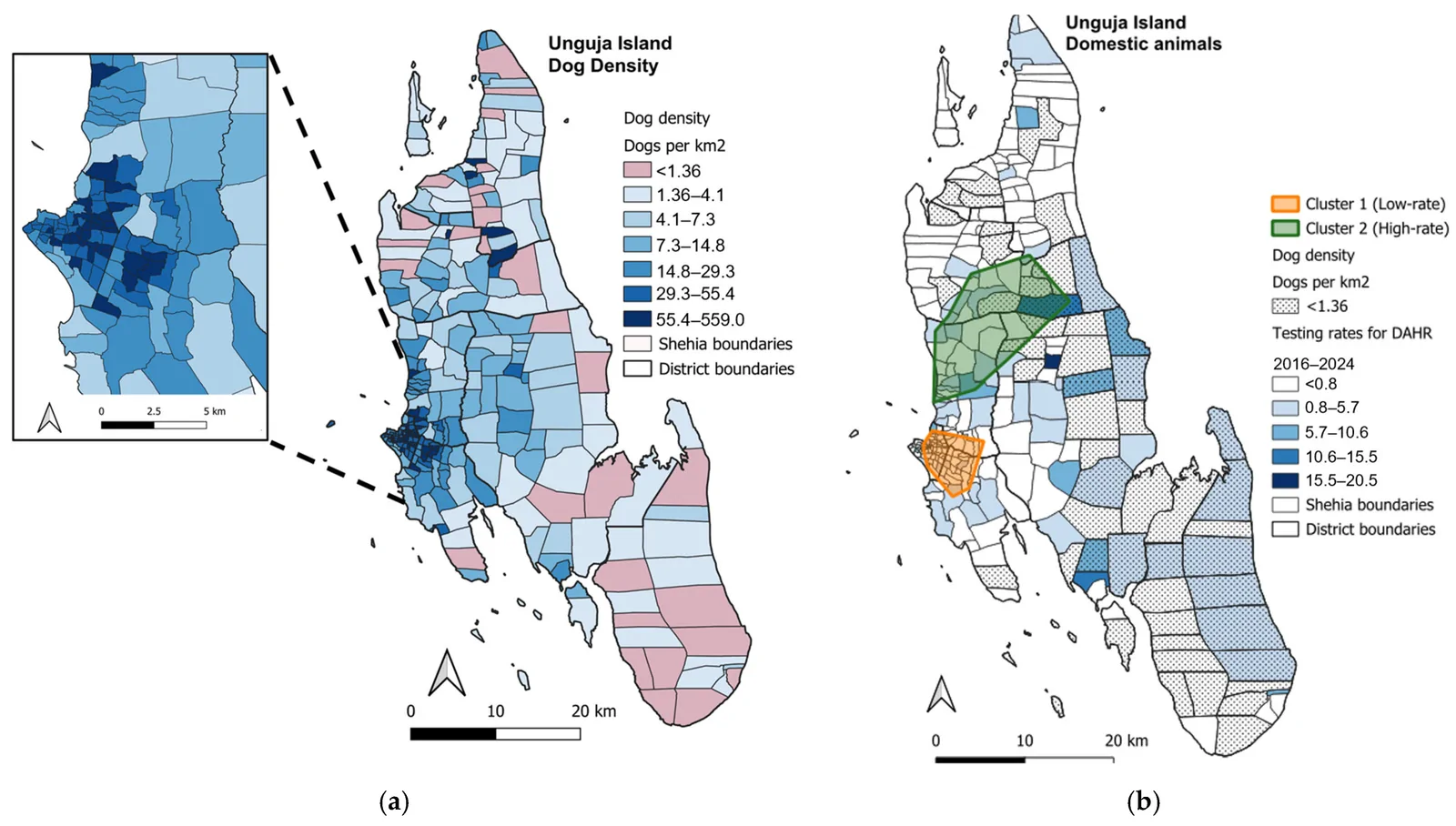
(**a**) Estimated dog density for each shehia on Unguja Island from the GARC technical report. (**b**) Significant disease clusters for domestic animals, with shehias DAHR and the shehias with dog density estimates below the threshold (shaded in black dots), for Unguja Island (2016–2024). This image was created by the author using QGIS Desktop v3.44.2.

After calculating the HDR for urban, rural and peri-urban shehia using the available dog population data from the GARC technical report, the dog density for shehias with unknown dog densities was estimated (Figure 11a). From this, the highest dog densities were found in the urban and mixed areas of the island (Mjini, Magharibi A and B), where the human population density is also high [23].

Only 29 shehias (12%) had a dog density below the threshold (<1.36 dogs per km^2^) to allow for rabies transmission, and can be found throughout Kaskazini A and B, Kusini and eastern regions of Kati. It is worth noting that these regions that are below the threshold are bordered by areas that have dog densities that are above the hypothetical transmission threshold. Figure 11b shows that both significant disease clusters for domestic animals are within a region that has a high enough dog density to sustain rabies transmission. Of the 195 shehias that were below the DAHR threshold, only 24 shehias have a dog density too low to maintain rabies transmission. Thus, 171 of these shehias have a below-threshold DAHR and a dog density high enough to sustain rabies transmission.

#### 3.2.5. Livestock and Wildlife Species

Although 26 samples (73% positivity rate) were submitted for livestock (Table 2) and 23 samples (30% positivity rate) for wildlife (Table 2), space–time analysis found no statistically significant disease clusters, and therefore livestock and wildlife were not further investigated.

## 4. Discussion

### 4.1. Molecular Epidemiology of RABV from Unguja Island and Africa

A molecular epidemiological analysis was undertaken to understand RABV transmission on Unguja Island in the broader context of Africa. From this analysis, seven genetically distinct clades were identified (clades A–G) circulating in southern and eastern regions in Africa. Clades A–F belong to the Africa 1b lineage first described by Kissi et al. [53], while clade G belongs to the Africa 1a lineage, which is prevalent in northern and western regions of Africa [53,54]. As all the newly generated sequences from Unguja Island grouped exclusively into clade D, only this clade will be discussed in further detail.

Clade D RABVs (Figure 2, Figure 3 and Figure 4) are spread over Unguja Island and other Tanzanian regions. The short branch lengths with the high posterior probability values indicate that the RABVs are genetically homogeneous and share a recent common ancestor (Figure 2). From this phylogenetic analysis, it appears that there are limited introductions of RABV from the mainland to Unguja Island, with sustained local maintenance of RABV in the dog population. In addition, this also shows the Unguja Island’s RABVs in relation to more genetically diverse African isolates.

Further looking at the sub-clades in clade D (Figure 4), sub-clade D-I was detected during 2017, while sub-clade D-II was detected from 2016 to 2017. Both these clades are restricted to the western urban regions of Unguja Island. During August 2017, GARC assisted in the implementation of strategic island-wide mass dog vaccination [37]. These vaccination efforts took place over a large area, including where these clades were present [9]. Thus, it seems likely that the short-lived transmission of RABV in these regions was due to vaccination efforts.

Sub-clade D-III (Figure 4) consists of RABVs from Unguja Island, Pemba Island and the Serengeti district of Tanzania (located in northern Tanzania) from 2009 to 2017. This shows the long-range movement of RABV-infected animals and reservoir species between the mainland and the Zanzibar Archipelago through human movement. Although no recent introduction into Unguja Island was seen as the isolates in this clade range from 2009 to 2017, rabies was introduced from the mainland, and this led to endemic cycles being established and maintained on the island. Although islands are seen as geographic barriers, this does not prevent the spread of RABV to these areas through human movement [45].

The distribution of sub-clade D-IV RABVs across Unguja Island, ranging from 2016 to 2024, suggests that there was no recent introduction of RABV into the island. Sub-clade D-IVa is spread over the central regions of Unguja Island and contains only isolates from domestic dogs from 2022 (Figure 4). This sub-clade covers a large geographic area, including areas with no reported rabies cases. In contrast, sub-clade D-IVb is widespread over Unguja Island and consists of different host species, including canines (Figure 4). Similar to other rabies-endemic countries, the lack of independent wildlife cycles demonstrated that domestic dogs are the main reservoir and are responsible for the sustained endemic transmission of RABV on Unguja Island, with spill-over to other species on the island [2,56].

Overall, sub-clade D-IV demonstrates that the dog population maintains rabies, and the free-roaming nature of most dogs on the island allows rabies to persist in the population through increased contact and connectivity between dogs [57]. Although Unguja Island is approximately 35 km away from the mainland, there is constant movement of humans and animals through ferry transport, which creates opportunities for the introduction of rabies to Unguja Island. Despite this, our data do not reflect any recent introduction to the island. Previous mass dog vaccination campaigns on Unguja Island between 2017 and 2018 vaccinated over 6000 dogs and 400 cats, reaching an estimated 50% vaccination coverage [11]. However, this did not reach the WHO-recommended 70% vaccination coverage threshold. Despite this, island-wide mass dog vaccination was implemented annually from 2020 to 2023, indicating continued efforts to control rabies on Unguja Island [37]. This shows that reaching and maintaining the 70% vaccination coverage is important to reduce the rabies cases and prevent the reintroduction of rabies into the population [4]. The absence of any new introductions to Unguja Island may be influenced by the limited movement of rabies-infected animals via ferries and boats coming from the mainland, however no data are available to confirm this. A quantitative risk assessment by Weng et al. [58] has shown in Taiwan that strict animal importation measures such as quarantine measures and other regulations are effective to prevent the reintroduction of rabies in a rabies-free area. Therefore, strengthening surveillance on Unguja Island will be important to determine if current control measures are sufficient in preventing the reintroduction of RABV.

For this molecular epidemiological analysis, the partial N gene was used. The coding N gene is conserved and therefore obtains fewer mutations, which allows the differentiation between closely related virus lineages; in turn, this allows the ancestry of RABVs to be determined [59,60]. Due to the homogeneous nature of the newly generated sequences from the RABVs in this study, a greater genetic resolution for these isolates could be obtained when utilizing whole-genome sequencing (WGS). However, no studies have reported using WGS from RNA extracted from an LFD. This is likely due to the low RNA concentration from an extracted nitrocellulose membrane, which could limit genome coverage. In addition, the higher costs and equipment associated with WGS may limit the feasibility in low-resource endemic areas. Because the RNA obtained from the LFDs in this study were possibly degraded due to the regulatory authority-mandated heat-inactivation step at the start of the RNA extraction process, another option is to utilize alternative individual genes or gene regions on the RABV genome using conventional PCR methods for molecular epidemiology.

### 4.2. The Use of the RAIDER Toolkit and Its Influence on the AAHR

As mentioned in the introduction, before the introduction of the RAIDER toolkit in 2022, rabies samples were largely collected using a passive surveillance system that relied on the DRIT (implemented in 2016) to diagnose suspected rabies cases [9,11]. Most of the cases originated from the urban, western side of the island where the ZCVL is closer in proximity. This centralized way of testing, while functional, had limited geographical coverage, and the RAIDER toolkit was implemented to improve surveillance on Unguja Island [13]. This toolkit, using LFDs for in-field diagnostic screening, was implemented to decentralize rabies testing, by improving testing in under-resourced areas that are unable to deliver suspected rabies samples to a diagnostic facility in a timely manner. It provides for the rapid detection of positive cases, which allows quicker responses such as PEP administration and targeted dog vaccination [13].

After the RAIDER toolkit was implemented, Moh’d et al. [13] investigated the impact of the LFDs on the surveillance system. From this study, it was found that sample submissions increased and continued increasing, with more samples being submitted from a bigger geographic area (more shehias) on the island. This resulted in more shehias having an above-threshold AAHR (>1.9), which shows an improvement in the surveillance coverage on Unguja Island (Figure 8).

An active surveillance program involves communities or healthcare workers actively looking for and reporting clinically suspect or probable rabid animals, and requires the testing of any suspected rabid animal, whether still alive or found dead. This leads to an increase in sample submission, and often results in an increased number of samples that tested negative, thus lowering the overall positivity rate while the number of samples tested increases [18]. This indicates whether sample testing in a surveillance network is considered adequate.

For the negative samples from this study, 46% of the sample submission reasons were “Animal was showing signs of rabies and humanely euthanized”, which could suggest an increased awareness of rabies signs among the inhabitants [13]. Despite the LFDs having this positive influence on increasing surveillance, sample submissions decreased in 2024 (Figure 5). This could be due to the resource-intensive and time-consuming nature of an active surveillance program, which is therefore harder to sustain in resource-limited endemic areas [61].

The findings from the present study extend those reported by Moh’d et al. [13]. While the previous study evaluated the impact of the RAIDER toolkit using surveillance data up to November 2023 and demonstrated increased sample submissions and geographical coverage, the present study included the full 2024 surveillance data together with shehia-level AAHR and DAHR analyses. These findings show that, although surveillance improved following the implementation of the RAIDER toolkit, sample submissions declined during 2024 and surveillance gaps remained, with many shehias still below the recommended surveillance thresholds. These findings highlight the need to sustain surveillance efforts to maintain and strengthen rabies surveillance coverage on Unguja Island.

### 4.3. SaTScan™ Analysis and Testing Rates

Space–time analysis using the Bernoulli model in SaTScan™ was utilized to identify disease hotspot areas (significant disease clusters) using all surveillance data, including both rabies-positive and rabies-negative cases. This enables data-driven decisions to be made for targeted strategies towards rabies elimination. Previous studies used the Discrete Poisson model on the SaTScan™ software (version 10.3.2) [35], which requires population data for the underlying population at risk [62,63,64,65], but this is difficult to use for dog populations because estimates based on human-to-dog ratios are often an underestimation of the true dog population numbers [66]. Given that there are no accurate dog population data available for Unguja Island, the proof-of-concept Bernoulli model developed by Malan et al. [7] was used to determine whether it could identify rabies hotspot clusters in a closed island environment where a fraction of rabies cases have been reported.

In these settings, simply considering the number of samples submitted per geographic area (whether in a table or plotted on a map) can be misleading, especially when sample submissions are low or uneven over the geographical region, as seen for the rabies surveillance data used in this study. SaTScan™ overcomes this limitation by statistically detecting disease clusters in areas where they do not visually appear to be a hotspot for rabies transmission (Figure 6 and Figure 9). These clusters are detected based on the RR, which compares the observed number of cases with the expected number of cases within a geographic area. Even when the sample submissions are low (the number of positive cases is low), when there are more observed cases than expected cases, the RR may be a high value. This should be interpreted with caution as the RR can be influenced by limited sample size, possibly due to inadequate surveillance coverage and distribution, rather than reflecting increased rabies transmission. This shows the benefit of using space–time analysis to identify areas that should be prioritized for enhanced surveillance and targeted public health interventions in regions with poor surveillance where disease hotspots might be overlooked.

#### 4.3.1. All Animals

Utilizing all the samples submitted for the entire study period (2016 to 2024), the space–time analysis found three significant disease clusters in all animal species on Unguja Island (Table 3 and Figure 6). To further determine if the lack of significant disease clusters in other areas on the island was due to the true absence of rabies cases or due to inadequate sampling, the AAHR were calculated using the same surveillance data. The AAHR across the island showed that only 24% of the shehias are above the threshold (AAHR > 1.9) (Figure 7). This shows inadequate surveillance over the entire island for all animal species.

This highlights the value of SaTScan™ in this surveillance dataset, where the geographical distribution does not always show transmission risk. When looking at the geographical distribution of the samples (Figure 6), some areas (such as cluster 1) have a large number of samples that will look like a hotspot when plotted on a map. But in comparison, cluster 2 and cluster 3 do not visually appear to be a hotspot. However, SaTScan™ analysis identified these areas as statistically significant, based on the RR.

Cluster 1, located in the urban western region of the island, was present after the implementation of the RAIDER toolkit from 2021 to 2024, and could still be active. This cluster had fewer observed cases than expected, showing a lower proportion of rabies-positive samples than expected. The low number of positive samples (*n* = 2) along with the high number of negative samples (*n* = 20) in the three districts that span cluster 1 (Figure 6) could suggest that the fewer observed cases may be due to disease control rather than poor surveillance in the cluster. This could be due to improved control efforts in this area, such as increased vaccinations, since this is a densely populated area for humans and dogs in relatively close proximity to the ZCVL (Figure 6 and Figure 11a). However, rabies vaccination coverage data were not used for comparison, which is a limitation for this study. Since clusters are detected based on the RR (calculated by the ratio of observed to expected cases), under-sampling and poor surveillance in the cluster, as shown by the below-threshold AAHR in large parts of cluster 1 and surrounding regions, could also influence cluster detection.

Increasing surveillance in the region of cluster 1 and in the surrounding areas may result in a smaller cluster. This is because, as more samples are submitted, which may possibly be mostly rabies-negative due to the vaccination efforts in the regions, an increase in negative cases detected both within and outside of this cluster will reduce the RR and statistical significance of the cluster, making the cluster smaller in size and geographic extent. This would reflect improved case detection rather than changes in the risk of rabies transmission. In contrast, if additional rabies-positive samples are identified, the RR and statistical significance may increase, potentially resulting in a larger cluster that covers a broader geographic region. Therefore, cluster 1 should be considered a candidate area for intensified surveillance rather than definitive evidence of reduced rabies transmission and successful disease control.

Cluster 2 and Cluster 3 were present from July 2016 to November 2020. These clusters could also have been present earlier and for longer, but due to the surveillance system only being implemented and operational in July 2016, no data is available to determine the true extent of these clusters, which could be underestimated.

Cluster 2, located in the central parts of the island, had more observed cases than expected, which indicates a higher proportion of rabies-positive samples submitted than expected in this area. Cluster 2 also includes a large region and surrounding areas that are below the AAHR threshold. Increasing surveillance efforts in this cluster and the surrounding areas could lead to more positive cases being detected. An increase in positive cases would result in a higher RR and increase the statistical significance, which would result in the cluster being bigger and covering a larger geographic area. The cluster could possibly expand further north, since a large area in Kaskazini Unguja region has below-threshold testing rates. In addition, this area is also further away from the ZCVL, which led to under-sampling, as discussed above. This clustering and the testing rates are similar to the situation described by Malan et al. [7] for the Eastern Cape (EC) province in SA, where low testing rates resulted in smaller and fewer clusters than they would have observed if surveillance were improved.

Cluster 3, found in the southern parts of the island, also has more observed cases than expected, and covers a larger area that has above-threshold and higher AAHR, but the surrounding areas are below the threshold. Overall, this cluster could also be larger and cover a larger geographic area, as with cluster 2, where changes in cluster size would reflect improved case detection rather than direct evidence of a change in rabies transmission. Therefore, clusters 2 and 3 should also be considered candidate areas for intensified surveillance. Enhanced surveillance within these clusters and the surrounding below-threshold areas may identify additional rabies cases, which would describe the true extent.

#### 4.3.2. Domestic Animals

Using SaTScan™ for the space–time analysis of all the samples for domestic animals over the entire study period (2016 to 2024), two statistically significant disease clusters were found (Figure 9). The DAHR across the island showed that only 26% of the shehias are above the threshold (DAHR > 0.8) (Figure 10), indicating inadequate surveillance.

The SaTScan™ analysis and DAHR for domestic animals (Figure 10) were similar to the All Animal analysis, with clusters 1 and 2 occurring in the same time frame and location. Although the Domestic Animals analysis provided similar results to All Animals, analyzing domestic animals separately remains important because they represent the rabies reservoir population on Unguja Island and Africa [2]. Livestock and wildlife species are generally incidental hosts, whereas domestic dogs sustain rabies transmission and are responsible for the majority of human rabies exposures [2]. The Domestic Animal analyses provide findings that are directly relevant to evaluating the surveillance system, which will drive control interventions. This shows that the continued prioritization of dog surveillance and vaccinations are important to achieve rabies elimination in the island.

Cluster 1 is smaller in geographic size compared to the All Animal cluster, which could indicate that domestic animals largely drove the observed space–time pattern seen in the All Animal cluster. This suggests that including livestock and wildlife slightly expanded the All Animal cluster. Apart from the cluster size, the same interpretations and conclusions can be made as discussed for All Animals (Section 4.3.1). Cluster 2 is almost identical in size to the All Animals cluster 2, which further indicates that dogs are responsible for maintaining rabies transmission in this area during this period.

These areas with low (AAHR > 1.9 and DAHR > 0.8) or below-threshold testing rates (AAHR < 1.9 and DAHR < 0.8) (Figure 7 and Figure 10) correspond with large areas that had no RABVs in the molecular analysis for clade D (Figure 4). This suggests that the absence of more isolates in this sub-clade could be due to poor surveillance in this area, rather than the true absence of RABV, demonstrating how improving surveillance coverage will also improve genomic surveillance.

#### 4.3.3. Dog Population Estimates

Dog population estimates are needed to understand rabies transmission dynamics. The high HDR estimates in this study (Table 1) indicate a small dog population of about 9865 dogs on Unguja Island (Table S4). Historical records reported the estimated dog population at 18,789 in 2008 and 9893 in 2013 [11]. The drastic decline in the dog population was attributed to an outbreak of Canine Distemper Virus (CDV) in 2010 and again in 2012 [10]. Based on 2017/2018 and 2020/2021 rabies vaccination data, the dog population was estimated at 10,552, and in the 2021/2022 vaccination data the dog population was estimated at 8611 [38]. These findings are similar to the number estimated in this study, suggesting that the dog populations possibly remained low since 2013 due to population decline, or more likely a recurring outbreak of CDV.

The predominantly Muslim population on Unguja Island also influences the low dog population numbers and the very high HDRs. A few studies in Chad found that a higher HDR was reported for Muslim households than Christian households, indicating that Muslim communities tend to own fewer dogs because they believe dogs are impure [67,68]. This was also shown in East Africa, including Ethiopia [69] and Tanzania [70], where Muslims keep fewer dogs, except when they are used for hunting, guarding, or security purposes in rural regions. This is reflected in the rural HDR (199.60) that is much lower than the urban HDR (356.13) (Table 1), which indicates more dogs per human in rural regions.

Dog density estimates were used in this study to determine where the dog density is theoretically high enough to maintain rabies transmission. Identifying areas with a below-threshold DAHR (<0.8) and dog density high enough to sustain rabies transmission (1.36 dog/km^2^) represents a surveillance gap on Unguja Island (Figure 11b). Both Clusters 1 and 2 for domestic animals have low DAHR and dog density high enough to sustain rabies transmission, or higher. Therefore, both significant disease clusters have poor surveillance and a high rabies transmission risk. This highlights the need for targeted surveillance in these areas to accurately determine the rabies burden, which will guide control interventions such as dog vaccinations.

Although only 12% of the shehias were below the DAHR threshold and dog density threshold needed to maintain rabies (Figure 11b), these shehias are surrounded by areas that have a high enough dog density to sustain rabies transmission. While animals in these shehias are theoretically at lower risk for rabies transmission, the shehias cannot be seen as isolated from rabies, since most of the dogs are free-roaming and not restricted by these administrative boundaries on Unguja Island, similar to other low-income regions [71]. Dogs can travel long distances when in the incubation phase of RABV infection, allowing RABV to spread over large areas [72], which further increases the potential for rabies transmission across the island. This shows that targeted surveillance needs to account for the movement of animals to accurately identify disease hotspots.

### 4.4. Study Limitations

This study has several limitations that should be considered when interpreting the results. The surveillance data consists of submitted samples; therefore, not all suspected rabies cases on Unguja Island may have been included. In addition, surveillance coverage varied across the island, with areas closer to the diagnostic laboratory submitting more samples, resulting in many shehias further away falling below the AAHR and DAHR thresholds, which indicates that the absence of disease clusters or molecularly characterized RABVs in some areas may be due to inadequate surveillance rather than the true absence of rabies. The SaTScan™ analysis was also based on the submitted surveillance data and may therefore underestimate the true extent of rabies transmission in areas with poor surveillance. Furthermore, vaccination coverage records were not included, and therefore the impact of vaccination on the identified disease clusters could not be confirmed. Despite these limitations, combining space–time cluster analysis with the AAHR and DAHR provided useful information to identify candidate areas for intensified surveillance, highlighting surveillance gaps to guide future surveillance and rabies control efforts on Unguja Island.

## 5. Conclusions

Rabies continues to pose a significant health concern in areas with limited surveillance capacities and resources, such as Unguja Island, where domestic dog populations are responsible for RABV transmission. Reliable surveillance is needed to inform the implementation of targeted control strategies and timely interventions, and to identify rabies hotspot areas. This study aimed to highlight the dual benefits of 1) using a diagnostic LFD for molecular epidemiology to enhance genomic surveillance and 2) assessing the rabies surveillance system using spatial analysis and mathematical evaluations to enhance rabies surveillance on Unguja Island.

Although previous studies have demonstrated the amplification and sequencing of RABV RNA from used LFDs, to our knowledge, this is the first time that the entire nitrocellulose membrane from an LFD was used successfully to extract RNA for further molecular epidemiological analysis. Our findings demonstrate that, in addition to the value of using LFDs in field settings as part of active surveillance, the samples from these LFDs can also be used to enhance genomic surveillance in resource-limited endemic areas. With WOAH acknowledging the potential value of LFDs and providing guidelines for their use, they can be implemented for rapid and low-cost diagnostic screening that limits the need for transporting brain samples, which is safer, more practical, and likely to enhance rabies surveillance.

Using a space–time analysis and testing rates, we detected significant disease clusters for all animals and domestic animals in areas with poor surveillance coverage where few cases were reported, indicating that these areas should be prioritized for strengthening surveillance. These findings suggest that the true extent of RABV transmission on Unguja Island may be underestimated due to uneven surveillance coverage. Although the implementation of the RAIDER toolkit has improved surveillance on Unguja Island, this study shows there are still gaps in the rabies surveillance system where surveillance needs to be increased to improve case detection, which will allow for more effective control measures and eventual freedom from rabies.

## Figures and Tables

**Table 1:** Estimated human-to-dog ratios calculated for the shehias on Unguja Island using the GARC technical report from 2022 and the human population data.

Ward Classification	Average Human-to-Dog Ratio
Urban	356.13
Rural	199.60
Mixed	277.86

**Table 2:** Rabies-positive and rabies-negative cases in Unguja Island per species group, 2016–2024.

Species	Negative Cases	Positive Cases	Total	% Positive Cases
Domestic animals	64	52	116	45%
Livestock	7	19	26	73%
Wildlife	16	7	23	30%
Total	87	78	165	47%

**Table 3:** Cluster information from SaTScan™ analysis for significant disease clusters for the entire dataset (2016 to 2024).

Cluster	Start Date	End Date	*p*-Value	*p*-Value	Observed Cases	Expected Cases	Relative Risk
1	2020/11/5	2024/11/4	0.00014	*p* < 0.001	2	14.182	0.118
2	2016/7/21	2020/11/4	0.00033	*p* < 0.001	17	8.036	2.426
3	2016/7/21	2020/11/4	0.00234	*p* < 0.01	15	7.091	2.381

**Table 4:** Rabies-positive and rabies-negative cases in Unguja Island for domestic animals, 2016–2024.

Species	Negative Cases	Positive Cases	% Positive Cases
Domestic dogs	41	41	50%
Domestic cats (*Felis catus*)	23	11	32%
Total	64	52	45%

**Table 5:** Cluster information from SaTScan™ analysis for significant disease clusters for domestic animals (2016 to 2024).

Cluster	Start Date	End Date	*p*-Value	*p*-Value	Observed Cases	Expected Cases	Relative Risk
1	2020/11/5	2024/11/4	0.00284	*p* < 0.05	0	7.621	0.000
2	2016/7/21	2020/11/4	0.01765	*p* < 0.05	11	4.931	2.561

## Data Availability

Human population data used in this study were obtained from the Tanzania 2022 Human Census data (https://www.nbs.go.tz/uploads/statistics/documents/en-1705482872-2019-20_Agri_Census_%20Main_Report.pdf, accessed on 15 April 2025). Estimated dog population data per shehia were derived from the 2022 Global Alliance for Rabies Control (GARC) technical reports (https://drive.google.com/file/d/1v1COCrgMLMrjsXK44VvZMjW-uTjG4NyI/view, accessed on 6 May 2025). Information about the area classification and size of the shehias was obtained from City Population (https://www.citypopulation.de/en/tanzania/zanzibar/admin/, accessed on 1 July 2025). Data used in the phylogenetic analysis are publicly available sequences and can be accessed on https://www.ncbi.nlm.nih.gov/genbank/ (accessed on 15 October 2025), according to the accession numbers. Surveillance data on rabies cases that support the findings of this study are available in the Supplementary Materials. The original contributions presented (through figures and tables) in the study are included in the article/Supplementary Materials, and further inquiries can be directed to the corresponding author.

## Supplementary Materials

The following supporting information can be downloaded at: https://www.mdpi.com/article/10.3390/v18080836/s1, Table S1: RABV-positive LFD samples used in this study from Unguja Island; Table S2: Sample set of RABVs included in the phylogenetic analysis of the partial N gene of Unguja Island and other African countries; Table S3: Unguja Island rabies surveillance data, July 2016–December 2024. Table S4: Dog density (dogs/km^2^) for all shehias in Unguja Island.

## Abbreviations

The following abbreviations are used in this manuscript:

AAHR	All-animal-per-human testing rate
ACGT	African Centre for Gene Technologies
AIC	Akaike’s Information Criterion
ARC-OVR	Agricultural Research Council—Onderstepoort Veterinary Research
BEAGLE	Broad-platform Evolutionary Analysis General Likelihood Evaluator
BEAST	Bayesian Evolutionary Analysis Sampling Trees
cDNA	Complementary DNA
CDV	Canine Distemper Virus
DALRRD	Department of Agriculture, Land Reform and Rural Development
DAHR	Domestic-animal-per-human testing rate
DD	Decimal degree
DFAT	Direct Fluorescent Antibody Test
DNA	Deoxyribonucleic acid
DRIT	Direct Rapid Immunohistochemical Test
EDTA	Ethylenediaminetetraacetic acid
EtOH	Ethanol
FABI	Forestry and Agricultural Biotechnology Institute
GARC	Global Alliance for Rabies Control
GPS	Global Positioning System
GTR + G	General Time Reversible model with Gamma distribution
H_2_O	Water
HDR	Human-to-dog ratio
km	Kilometre
km^2^	Square kilometre
LFD	Lateral flow device
MAINL	Ministry of Agriculture, Irrigation, Natural Resource and Livestock
MCMC	Markov Chain Monte Carlo
MEGA11	Molecular Evolutionary Genetics Analysis version 11
min	Minute
ml	Milliliter
NaOAc	Sodium acetate
NCBI	National Center for Biotechnology Information
nt	Nucleotides
PEP	Post-exposure prophylaxis
PCR	Polymerase chain reaction
PoC	Point of Care
Q1	First quartile
QGIS	Quantum Geographic Information System
RABV	Rabies virus
RAIDER	Rapid In-field Diagnosis and Epidemiology of Rabies
RCS	Rabies Case Surveillance
RNA	Ribonucleic acid
RR	Relative risk
SA	South Africa
sec	Second
TAD-P	Transboundary Animal Diseases Programme
URT	United Republic of Tanzania
USA	United States of America
WAP	World Animal Protection
WGS	Whole genome sequencing
WHO	World Health Organization
ZCVL	Zanzibar Central Veterinary Laboratory
