# Supplementary material for: Using Lateral Flow Devices to Enhance Molecular Epidemiology and Evaluating the Rabies Surveillance System on Unguja Island, Zanzibar
Source: Viruses. 2026 Jul 29;18(8):836. doi: 10.3390/v18080836 (PMC13517439; doi:10.3390/v18080836)
Supplement: Supplementary file 1 [file viruses-18-00836-s001.zip › viruses-4404240-supplementary.pdf]

Table S1: RABV-positive LFD samples used in this study from Unguja Island.

Virus identification number	**Year**	**Species isolated from**	**District**	**Shehia**	**Latitude**	**Longitude**	**Accession number**
**ZANdog1/22**	2022	Canine	Kati	Pongwe	-6.05346	39.40976	PX458845
**ZANdog10/22**	2022	Canine	Kati	Ndijanimseweni	-6.19012	39.35202	PX458846
**ZANdog11/22**	2022	Canine	Kati	Ndijanimseweni	-6.19012	39.35202	PX458847
**ZANdog20/22**	2022	Canine	Magharibi A	Kibweni	-6.11466	39.21887	PX458848
**ZANdog21/22**	2022	Canine	Magharibi B	Maungani	-6.23333	39.25000	PX458849
**ZANdog29/22**	2022	Canine	Kati	Ndijanimseweni	-6.19194	39.34500	PX458850
**ZANcat30/22**	2022	Feline	Mjini	Kikwajuni Bondeni	-6.16672	39.19441	PX458851
**ZANdog33/22**	2022	Canine	Kati	Ndijanimseweni	-6.19194	39.34500	PX458852
**ZANmon6/23**	2023	Vervet monkey	Kati	Tunguu	-6.20000	39.31667	PX458853
**ZANbov8/23**	2023	Bovine	Kaskazini B	Kilombero	-6.02435	39.32661	PX458854
**ZANdog9/23**	2023	Canine	Kaskazini B	Kilombero	-6.02435	39.32661	PX458855
**ZANdog10/23**	2023	Canine	Magharibi A	Mtoni	-6.12644	39.21553	PX458856
**ZANdog12/23**	2023	Canine	Magharibi B	Uwandani	-6.20761	39.27341	PX458857
**ZANdog16/23**	2023	Canine	Magharibi B	Kiembesamaki	-6.20967	39.20316	PX458858
**ZANdog17/23**	2023	Canine	Kaskazini B	Kilombero	-6.02021	39.32333	PX458859
**ZANdog18/23**	2023	Canine	Kati	Jumbi	-6.20114	39.29741	PX458860
**ZANdog20/23**	2023	Canine	Kusini	Kitogani	-6.27090	39.42809	PX458861
**ZANcat28/23**	2023	Feline	Kusini	Paje	-6.27495	39.53419	PX458862
**ZANcat46/23**	2023	Feline	Kati	Tindini	-6.29426	39.36681	PX458863
**ZANdog47/23**	2023	Canine	Kati	Tindini	-6.29426	39.36968	PX458864
**ZANdog1/24**	2024	Dog	Magharibi A	Mfenesini	-6.05003	39.22428	PX458865
**ZANbov2/24**	2024	Bovine	Magharibi A	Mwera	-6.15796	39.26299	PX458866
**ZANlcap8/24**	2024	Caprine	Magharibi B	Dimani	-6.27645	39.27641	PX458867
**ZANcap11/24**	2024	Caprine	Magharibi B	Fumba	-6.30915	39.27764	PX458868

Table S2: Sample set of RABVs included in the phylogenetic analysis of the partial N gene of Unguja Island and other African countries.

Year	Sample number	Species	Country	Province	District	Latitude	Longitude	Clade	Sub-clade	Accession number	Authors
1986	86031MOZ	Canine	Mozambique	Unknown	Unknown	Unknown	Unknown	A	A-V	KX148203	Troupin *et al.*[1]
1990	RV390	Canine	Botswana	--	North-East (Francistown)	Unknown	Unknown	A	A-I	AY330738	Johnson *et al.*[2]
1994	22759	African civet	Zimbabwe	Mashonaland Central	Shamva	Unknown	Unknown	A	A-III	KY553271	Sabeta *et al.*[3]
1994	22319	African civet	Zimbabwe	Mashonaland Central	Concession	Unknown	Unknown	A	A-I	KY553270	Sabeta *et al.*[3]
2000	ZAMRAV31	Canine	Zambia	Unknown	Unknown	Unknown	Unknown	B	B	AB284507	Unpublished
2004	567/04	Canine	South Africa	Mpumalanga	Ehlanzeni	-26	30	A	A-V	HM179505	Kgaladi *et al.*[4]
2005	ZAMRAV09/05	Canine	Zambia	Unknown	Unknown	Unknown	Unknown	B	B	AB284508	Unpublished
2008	UPV130	Canine	South Africa	KwaZulu-Natal	Umkhanyakude	-27.62236	32.32945	A	A-IV	JF747614	Kgaladi *et al.*[4]
2009	RV2503	Wild Cat	Tanzania	Mara	Serengeti	-2.15999839	34.67903027	D	D-III	KR906740	Brunker *et al.*[5]
2010	RV2770	Canine	Tanzania	Dar es Salaam	Kiamboni	-6.88232192	39.38749264	F	F	KR906743	Brunker *et al.*[5]
2010	RV2771	Canine	Tanzania	Iringa	Mufindi	-8.55803262	35.27626447	F	F	KR906744	Brunker *et al.*[5]
2010	RV2807	Unknown species	Tanzania	Lindi	Nachingwea	-10.3998164	38.36771925	A	A-II	KR906757	Brunker *et al.*[5]
2010	RV2815	Bovine	Tanzania	Morogoro	Kilombero	-8.08945524	36.66729976	C	C	KR906764	Brunker *et al.*[5]
2010	RV2817	Canine	Tanzania	Kusini Pemba	Chake Chake	-5.26091486	39.81501568	C	C	KR906766	Brunker *et al.*[5]
2010	RV2773	Canine	Tanzania	Pwani	Kibaha	-6.78441261	38.76459085	E	E	KR906745	Brunker *et al.*[5]
2011	RV2780	Unknown species	Tanzania	Lindi	Nachingwea	-10.3998164	38.36771925	A	A-II	KR906751	Brunker *et al.*[5]
2011	RV2862	Canine	Tanzania	Mara	Serengeti	-1.75734405	34.56655744	D	D-III	KR906768	Brunker *et al.*[5]
2011	RV2907	Bovine	Tanzania	Mara	Serengeti	-1.77484421	34.80294235	F	F	KR906776	Brunker *et al.*[5]
2012	RV2767	Canine	Tanzania	Mara	Serengeti	-2.01628201	34.4851594	D	D-III	KR906742	Brunker *et al.*[5]
2012	RV2778	Canine	Pemba Island, Tanzania	Kusini Pemba	Chake Chake	-5.26091486	39.81501568	D	D-III	KR906750	Brunker *et al.*[5]
2012	RV3128	Donkey	Tanzania	Mara	Serengeti	-1.83521881	34.56841239	E	E	KR906786	Brunker *et al.*[5]
2016	RAB16024/ D07299SERIAL4VIR OLOGY29	Canine	Pemba Island, Tanzania	Kaskazini Pemba	Wete	Unknown	Unknown	D	D-III	OP375411	Lushasi *et al.*[6]
2016	ZANdog08/16	Canine	Unguja Island, Tanzania	Kaskazini Unguja	Kaskazini B	-5.9697735	39.3089485	D	D-IV_II	MG407529	Unpublished

Year	Sample number	Species	Country	Province	District	Latitude	Longitude	Clade	Sub-clade	Accession number	Authors
2016	ZANcap09/16	Caprine	Unguja Island, Tanzania	Mjini Magharibi	Magharibi B	-6.2051964	39.2479409	D	D-III	MG407530	Unpublished
2016	ZANdog10/16	Canine	Unguja Island, Tanzania	Kusini Unguja	Kusini	-6.4129936	39.5523859	D	D-III	MG407531	Unpublished
2016	ZANcap11/16	Caprine	Unguja Island, Tanzania	Mjini Magharibi	Mjini	-6.162222	39.1920735	D	D-III	MG407532	Unpublished
2016	ZANfel19/16	Feline	Unguja Island, Tanzania	Mjini Magharibi	Mjini	-6.165917	39.202641	D	D-III	MG407533	Unpublished
2016	ZANfel20/16	Feline	Unguja Island, Tanzania	Mjini Magharibi	Mjini	-6.1630556	39.22	D	D-II	MG407534	Unpublished
2016	ZANdog24/16	Canine	Unguja Island, Tanzania	Kusini Unguja	Kati	-6.1333333	39.3	D	D-IV_II	MG407535	Unpublished
2016	ZANdog25/16	Canine	Unguja Island, Tanzania	Kaskazini Unguja	Kaskazini B	-6.0084272	39.2401484	D	D-IV_II	MG407536	Unpublished
2016	RAB16026/ SD560	Canine	Pemba Island, Tanzania	Kaskazini Pemba	Wete	Unknown	Unknown	D	D-III	OP375417	Lushasi *et al.*[6]
2016	RAB16027	Canine	Pemba Island, Tanzania	Kusini Pemba	Chake Chake	Unknown	Unknown	D	D-III	OP375408	Lushasi *et al.*[6]
2016	RAB16028	Canine	Pemba Island, Tanzania	Kaskazini Pemba	Micheweni	Unknown	Unknown	D	D-III	OP375418	Lushasi *et al.*[6]
2016	RAB16029	Canine	Pemba Island, Tanzania	Kusini Pemba	Mkoani	Unknown	Unknown	D	D-III	OP375413	Lushasi *et al.*[6]
2017	ZANequ01/17	Equine	Unguja Island, Tanzania	Kusini Unguja	Kati	-6.2051964	39.3479409	D	D-III	MG407537	Unpublished
2017	ZANfel02/17	Feline	Unguja Island, Tanzania	Mjini Magharibi	Magharibi A	-6.1002778	39.22666667	D	D-I	MG407538	Unpublished
2017	ZANdog03/17	Canine	Unguja Island, Tanzania	Mjini Magharibi	Magharibi B	-6.0322222	39.22638889	D	D-I	MG407539	Unpublished
2017	ZANpri04/17	Primate	Unguja Island, Tanzania	Kusini Unguja	Kusini	-6.2415572	39.4139503	D	D-III	MG407540	Unpublished
2017	ZANcap13/17	Caprine	Unguja Island, Tanzania	Kusini Unguja	Kusini	-6.3158921	39.5446313	D	D-III	MG407541	Unpublished
2017	ZANdog16/17	Canine	Unguja Island, Tanzania	Kusini Unguja	Kusini	-6.4	39.55	D	D-III	MG407542	Unpublished
2017	ZANdog17/17	Canine	Unguja Island, Tanzania	Mjini Magharibi	Magharibi B	-6.1961548	39.2297569	D	D-III	MG407543	Unpublished
2017	ZANfel18/17	Feline	Unguja Island, Tanzania	Mjini Magharibi	Mjini	-6.1586111	39.20638889	D	D-I	MG407544	Unpublished
2017	ZANcap27/17	Caprine	Unguja Island, Tanzania	Kusini Unguja	Kusini	-6.3847222	39.54	D	D-III	MG407545	Unpublished

Year	Sample number	Species	Country	Province	District	Latitude	Longitude	Clade	Sub-clade	Accession number	Authors
2017	ZANcap28/17	Caprine	Unguja Island, Tanzania	Kusini Unguja	Kusini	-6.3847222	39.54	D	D-III	MG407546	Unpublished
2017	ZANfel29/17	Feline	Unguja Island, Tanzania	Mjini Magharibi	Mjini	-6.1630556	39.22	D	D-I	MG407547	Unpublished
2017	ZANdog30/17	Canine	Unguja Island, Tanzania	Mjini Magharibi	Mjini	-6.1333333	39.3	D	D-III	MG407548	Unpublished
2017	ZANfel31/17	Feline	Unguja Island, Tanzania	Kaskazini Unguja	Kaskazini B	-6.0166667	39.25	D	D-IV_II	MG407549	Unpublished
2017	ZANdog32/17	Canine	Unguja Island, Tanzania	Kaskazini Unguja	Kaskazini B	-6.10629	39.22932	D	D-I	MG407550	Unpublished
2017	ZANfel33/17	Feline	Unguja Island, Tanzania	Mjini Magharibi	Mjini	-6.1630556	39.20694444	D	D-II	MG407551	Unpublished
2017	ZANdog34/17	Canine	Unguja Island, Tanzania	Mjini Magharibi	Magharibi A	-6.1333333	39.25	D	D-I	MG407552	Unpublished
2017	LPdog307/17	Canine	South Africa	Limpopo	Capricorn	-23.4667	29.7	A	A-III	MW343955	Unpublished
2018	368/18 MOZ/Nampula	Canine	Mozambique	Nampula	Unknown	Unknown	Unknown	A	A-V	MW248384	Mapatse *et al.*[7]
2018	124/18 MOZ/Maputo	Canine	Mozambique	Maputo	Unknown	Unknown	Unknown	A	A-IV	MW248387	Mapatse *et al.*[7]
2019	NWdog191/19	Canine	South Africa	North West	Bojanala Platinum	-25.6676	27.24208	A	A-V	MW343973	Malan *et al.*[8]
2019	LPdog197/19	Canine	South Africa	Limpopo	Vhembe	-23.0439	29.9032	A	A-III	MW343916	Unpublished
2020	Z0083192	Canine	Kenya	Makueni county	--	-1.786495	37.631107	E	E	OR045951	Jaswant *et al.*[9]
2021	Z00861841	Canine	Kenya	Siaya	--	-0.0594856	34.2839401	G	G-II	OR045984	Jaswant *et al.*[9]
2022	**ZANdog1/22**	Canine	Unguja Island, Tanzania	Kusini Unguja	Kati	-6.05346	39.40976	D	D-IV_I	PX458845	This study
2022	**ZANdog10/22**	Canine	Unguja Island, Tanzania	Kusini Unguja	Kati	-6.19012	39.35202	D	D-IV_II	PX458846	This study
2022	**ZANdog11/22**	Canine	Unguja Island, Tanzania	Kusini Unguja	Kati	-6.19012	39.35202	D	D-IV_I	PX458847	This study
2022	**ZANdog20/22**	Canine	Unguja Island, Tanzania	Mjini Magharibi	Magharibi A	-6.11466	39.21887	D	D-IV_I	PX458848	This study
2022	**ZANdog21/22**	Canine	Unguja Island, Tanzania	Mjini Magharibi	Magharibi B	-6.23333	39.25000	D	D-IV_II	PX458849	This study
2022	**ZANdog29/22**	Canine	Unguja Island, Tanzania	Kusini Unguja	Kati	-6.19194	39.34500	D	D-IV_II	PX458850	This study
2022	**ZANcat30/22**	Feline	Unguja Island, Tanzania	Mjini Magharibi	Mjini	-6.16672	39.19441	D	D-IV_II	PX458851	This study
2022	**ZANdog33/22**	Canine	Unguja Island, Tanzania	Kusini Unguja	Kati	-6.19194	39.34500	D	D-IV_II	PX458852	This study

Year	Sample number	Species	Country	Province	District	Latitude	Longitude	Clade	Sub-clade	Accession number	Authors
2022	Z00865564	Canine	Kenya	Siaya	Rarieda	-0.1400055	34.3601107	G	G-II	OR046012	Jaswant *et al.*[9]
2023	**ZANmon6/23**	Vervet monkey	Unguja Island, Tanzania	Kusini Unguja	Kati	-6.20000	39.31667	D	D-IV_II	PX458853	This study
2023	**ZANbov8/23**	Bovine	Unguja Island, Tanzania	Kaskazini Unguja	Kaskazini B	-6.02435	39.32661	D	D-IV_II	PX458854	This study
2023	**ZANdog9/23**	Canine	Unguja Island, Tanzania	Kaskazini Unguja	Kaskazini B	-6.02435	39.32661	D	D-IV_II	PX458855	This study
2023	**ZANdog10/23**	Canine	Unguja Island, Tanzania	Mjini Magharibi	Magharibi A	-6.12644	39.21553	D	D-IV_II	PX458856	This study
2023	**ZANdog12/23**	Canine	Unguja Island, Tanzania	Mjini Magharibi	Magharibi B	-6.20761	39.27341	D	D-IV_II	PX458857	This study
2023	**ZANdog16/23**	Canine	Unguja Island, Tanzania	Mjini Magharibi	Magharibi B	-6.20967	39.20316	D	D-IV_II	PX458858	This study
2023	**ZANdog17/23**	Canine	Unguja Island, Tanzania	Kaskazini Unguja	Kaskazini B	-6.02021	39.32333	D	D-IV_II	PX458859	This study
2023	**ZANdog18/23**	Canine	Unguja Island, Tanzania	Kusini Unguja	Kati	-6.20114	39.29741	D	D-IV_II	PX458860	This study
2023	**ZANdog20/23**	Canine	Unguja Island, Tanzania	Kusini Unguja	Kusini	-6.27090	39.42809	D	D-IV_II	PX458861	This study
2023	**ZANcat28/23**	Feline	Unguja Island, Tanzania	Kusini Unguja	Kusini	-6.27495	39.53419	D	D-IV_II	PX458862	This study
2023	**ZANcat46/23**	Feline	Unguja Island, Tanzania	Kusini Unguja	Kati	-6.29426	39.36681	D	D-IV_II	PX458863	This study
2023	**ZANdog47/23**	Canine	Unguja Island, Tanzania	Kusini Unguja	Kati	-6.29426	39.36968	D	D-IV_II	PX458864	This study
2024	**ZANdog1/24**	Canine	Unguja Island, Tanzania	Mjini Magharibi	Magharibi A	-6.05003	39.22428	D	D-IV_II	PX458865	This study
2024	**ZANbov2/24**	Bovine	Unguja Island, Tanzania	Mjini Magharibi	Magharibi A	-6.15796	39.26299	D	D-IV_II	PX458866	This study
2024	**ZANcap8/24**	Caprine	Unguja Island, Tanzania	Mjini Magharibi	Magharibi B	-6.27645	39.27641	D	D-IV_II	PX458867	This study
2024	**ZANcap11/24**	Caprine	Unguja Island, Tanzania	Mjini Magharibi	Magharibi B	-6.30915	39.27764	D	D-IV_II	PX458868	This study
2020 (2019)	20RD16	*Mus musculus* (Human)	Italy (Unguja Island)	Unknown	Unknown	Unknown	Unknown	D	D-IV_II	OQ787037	Castellan *et al.*[10]
Unknown	ETH8	Unknown species	Ethiopia	Unknown	Unknown	Unknown	Unknown	G	G-I	FJ947032	Marston *et al.*[11]
Unknown	Rv520	Jackal	Ethiopia	Unknown	Unknown	Unknown	Unknown	G	G-I	AY502132	Johnson *et al.*[12]

Year	Sample number	Species	Country	Province	District	Latitude	Longitude	Clade	Sub-clade	Accession number	Authors
Unknown	RV521	Jackal	Ethiopia	Unknown	Unknown	Unknown	Unknown	G	G-I	AY103015	Johnson *et al.*[12]
	Pasteur Virus					Unknown	Unknown	Outgroup		M13215	Tordo *et al.*[13]

Table S3: Unguja Island rabies surveillance data, July 2016 - December 2024.

#	Sample number	Date	Binned Species	Species	Province	District	Shehia	Result	Latitude	Longitude
1	010/16	2016-07-21	Domestic animals	Canine	Kusini Unguja	Kusini	Kiongoni	Positive	-6.41299	39.55239
2	011/16	2016-07-27	Livestock	Caprine	Mjini Magharibi	Mjini	Mkunazini	Positive	-6.16222	39.19207
3	012/16	2016-07-28	Wildlife	Rodent	Mjini Magharibi	Mjini	Rahaleo	Negative	-6.16403	39.20182
4	013/16	2016-08-03	Livestock	Bovine	Mjini Magharibi	Mjini	Maruhubi	Negative	-6.14708	39.21020
5	019/16	2016-09-30	Domestic animals	Feline	Mjini Magharibi	Mjini	Muembeshauri	Positive	-6.16592	39.20264
6	020/16	2016-10-13	Domestic animals	Feline	Mjini Magharibi	Mjini	Amani	Positive	-6.16306	39.22000
7	024/16	2016-11-05	Domestic animals	Canine	Kusini Unguja	Kati	Kidimni	Positive	-6.13333	39.30000
8	025/16	2016-11-29	Domestic animals	Canine	Kaskazini Unguja	Kaskazini B	Matetema	Positive	-6.00843	39.24015
9	026/16	2016-12-17	Domestic animals	Feline	Mjini Magharibi	Magharibi B	Fuoni Kipungani	Negative	-6.20520	39.24794
10	001/17	2017-01-06	Livestock	Equine	Kusini Unguja	Kati	Ndijanimseweni	Positive	-6.20520	39.34794
11	002/17	2017-01-09	Domestic animals	Feline	Mjini Magharibi	Magharibi A	Kijichi	Positive	-6.10028	39.22667
12	003/17	2017-01-17	Domestic animals	Canine	Kaskazini Unguja	Kaskazini B	Mkadini	Positive	-6.03222	39.22639
13	004/17	2017-02-16	Wildlife	Wildlife - Vervet monkey	Kusini Unguja	Kati	Charawe	Positive	-6.24156	39.41395
14	008/17	2017-02-28	Wildlife	Wildlife - Vervet monkey	Kaskazini Unguja	Kaskazini A	Mtakuja	Negative	-5.81861	39.22310
15	009/17	2017-02-28	Wildlife	Wildlife - Vervet monkey	Kaskazini Unguja	Kaskazini A	Mtakuja	Negative	-5.81861	39.22310
16	010/17	2017-02-28	Wildlife	Wildlife - Vervet monkey	Kaskazini Unguja	Kaskazini A	Mtakuja	Negative	-5.81861	39.22310
17	011/17	2017-02-28	Wildlife	Wildlife - Vervet monkey	Kaskazini Unguja	Kaskazini A	Mtakuja	Negative	-5.81861	39.22310
18	012/17	2017-02-28	Wildlife	Wildlife - Vervet monkey	Kaskazini Unguja	Kaskazini A	Mtakuja	Negative	-5.81861	39.22310
19	013/17	2017-03-02	Livestock	Caprine	Kusini Unguja	Kusini	Jambiani Kibigija	Positive	-6.31589	39.54463
20	016/17	2017-04-04	Domestic animals	Canine	Kusini Unguja	Kusini	Kajengwa	Positive	-6.40000	39.55000
21	017/17	2017-05-19	Domestic animals	Canine	Mjini Magharibi	Magharibi B	Tomondo	Positive	-6.19616	39.22976
22	018/17	2017-05-19	Domestic animals	Feline	Mjini Magharibi	Mjini	Mitiulaya	Positive	-6.15861	39.20639
23	027/17	2017-06-09	Livestock	Caprine	Kusini Unguja	Kusini	Kajengwa	Positive	-6.38472	39.54000
24	028/17	2017-06-09	Livestock	Caprine	Kusini Unguja	Kusini	Kajengwa	Positive	-6.38472	39.54000
25	029/17	2017-06-29	Domestic animals	Feline	Mjini Magharibi	Mjini	Amani	Positive	-6.16306	39.22000

#	Sample number	Date	Binned Species	Species	Province	District	Shehia	Result	Latitude	Longitude
26	030/17	2017-07-30	Domestic animals	Canine	Kusini Unguja	Kati	Kidimni	Positive	-6.13333	39.30000
27	031/17	2017-08-05	Domestic animals	Feline	Kaskazini Unguja	Kaskazini B	Kitope	Positive	-6.01667	39.25000
28	032/17	2017-08-05	Domestic animals	Canine	Mjini Magharibi	Magharibi A	Chemchem	Positive	-6.10629	39.22932
29	033/17	2017-08-10	Domestic animals	Feline	Mjini Magharibi	Mjini	Mikunguni	Positive	-6.16306	39.20694
30	034/17	2017-08-10	Domestic animals	Canine	Mjini Magharibi	Magharibi A	Kianga	Positive	-6.13333	39.25000
31	039/17	2017-08-10	Livestock	Caprine	Mjini Magharibi	Magharibi B	Kombeni	Positive	-6.24889	39.27444
32	040/17	2017-08-10	Livestock	Caprine	Mjini Magharibi	Magharibi B	Kombeni	Positive	-6.24889	39.27444
33	041/17	2017-12-04	Domestic animals	Canine	Kaskazini Unguja	Kaskazini A	Kinyasini	Positive	-5.96977	39.30895
34	042/17	2017-12-04	Domestic animals	Canine	Kaskazini Unguja	Kaskazini A	Kinyasini	Positive	-5.96977	39.30895
35	043/17	2017-12-04	Livestock	Caprine	Mjini Magharibi	Magharibi A	Chuini	Positive	-6.06528	39.22083
36	001/18	2018-01-18	Domestic animals	Canine	Kusini Unguja	Kati	Miwani	Positive	-6.07750	39.28472
37	002/18	2018-02-28	Domestic animals	Canine	Kusini Unguja	Kati	Cheju	Positive	-6.21472	39.37861
38	003/18	2018-02-28	Livestock	Caprine	Mjini Magharibi	Magharibi B	Maungani	Positive	-6.22944	39.25306
39	004/18	2018-04-23	Livestock	Caprine	Mjini Magharibi	Magharibi B	Maungani	Positive	-6.22944	39.25306
40	005/18	2018-04-25	Domestic animals	Canine	Mjini Magharibi	Magharibi A	Sharifumsa	Positive	-6.11806	39.21083
41	006/18	2018-06-28	Domestic animals	Canine	Mjini Magharibi	Magharibi A	Kianga	Positive	-6.12889	39.25389
42	007/18	2018-07-12	Domestic animals	Canine	Mjini Magharibi	Magharibi A	Mwera	Positive	-6.15778	39.26278
43	008/18	2018-07-31	Domestic animals	Canine	Mjini Magharibi	Magharibi A	Mfenesini	Positive	-6.04611	39.23222
44	001/19	2019-01-08	Wildlife	Wildlife - Red colobus monkey	Kusini Unguja	Kati	Pete	Positive	-6.28245	39.40916
45	002/19	2017-07-24	Domestic animals	Canine	Mjini Magharibi	Magharibi B	Kisauni	Positive	-6.21700	39.22852
46	003/19	2019-11-21	Domestic animals	Canine	Kusini Unguja	Kusini	Dongwe	Positive	-6.23724	39.53232
47	004/19	2019-12-14	Domestic animals	Canine	Kaskazini Unguja	Kaskazini B	Kiwengwa	Negative	-5.99010	39.37608
48	005/19	2019-12-16	Livestock	Bovine	Mjini Magharibi	Magharibi A	Kizimbani	Positive	-6.08333	39.26667
49	006/19	2019-12-24	Domestic animals	Canine	Kaskazini Unguja	Kaskazini B	Mkataleni	Positive	-5.98834	39.24995
50	007/19	2019-12-24	Domestic animals	Canine	Kusini Unguja	Kusini	Michamvi	Positive	-6.15000	39.50000
51	008/19	2019-12-24	Wildlife	Wildlife - Vervet monkey	Mjini Magharibi	Magharibi A	Kijichi	Positive	-6.09886	39.22045
52	009/19	2019-12-26	Livestock	Caprine	Kaskazini Unguja	Kaskazini B	Mkataleni	Positive	-5.98834	39.24995

#	Sample number	Date	Binned Species	Species	Province	District	Shehia	Result	Latitude	Longitude
53	010/19	2019-12-30	Wildlife	Wildlife - Vervet monkey	Kusini Unguja	Kusini	Jambiani Kibigija	Positive	-6.32189	39.54679
54	001/20	2020-01-23	Wildlife	Wildlife - Vervet monkey	Kusini Unguja	Kusini	Jambiani Kibigija	Positive	-6.32189	39.54679
55	002/20	2020-02-18	Livestock	Caprine	Mjini Magharibi	Magharibi A	Kijichi	Positive	-6.09886	39.22045
56	003/20	2020-05-04	Livestock	Caprine	Mjini Magharibi	Magharibi A	Kijichi	Positive	-6.09886	39.22045
57	004/20	2020-07-02	Domestic animals	Feline	Mjini Magharibi	Magharibi B	Uwandani	Positive	-6.19695	39.26435
58	001/21	2021-01-04	Livestock	Caprine	Mjini Magharibi	Magharibi A	Mwera	Positive	-6.14629	39.25853
59	002/21	2021-01-21	Wildlife	Wildlife - Vervet monkey	Kusini Unguja	Kusini	Kiongoni	Positive	-6.41275	39.55337
60	003/21	2021-01-26	Domestic animals	Canine	Kaskazini Unguja	Kaskazini A	Kigunda	Positive	-5.77810	39.29982
61	006/21	2021-02-23	Domestic animals	Canine	Mjini Magharibi	Magharibi A	Kijichi	Positive	-6.09886	39.22045
62	007/21	2021-04-09	Domestic animals	Canine	Kusini Unguja	Kati	Chwaka	Negative	-6.16521	39.43513
63	008/21	2021-04-20	Domestic animals	Canine	Mjini Magharibi	Magharibi A	Kianga	Positive	-6.13095	39.25373
64	009/21	2021-06-28	Domestic animals	Feline	Mjini Magharibi	Magharibi B	Sokoni	Negative	-6.17734	39.22329
65	010/21	2021-06-28	Domestic animals	Canine	Mjini Magharibi	Mjini	Mikunguni	Negative	-6.16339	39.20715
66	011/21	2021-07-25	Livestock	Caprine	Mjini Magharibi	Magharibi A	Kijichi	Negative	-6.09886	39.22045
67	012/21	2021-11-26	Domestic animals	Canine	Kaskazini Unguja	Kaskazini A	Mkwajuni	Positive	-5.88626	39.28715
68	001/22	2022-03-10	Domestic animals	Canine	Kusini Unguja	Kati	Pongwe	Positive	-6.05346	39.40976
69	002/22	2022-03-14	Domestic animals	Feline	Mjini Magharibi	Mjini	Kilimani (Mjini)	Negative	-6.18334	39.20533
70	003/22	2022-04-20	Domestic animals	Feline	Mjini Magharibi	Magharibi A	Kianga	Negative	-6.12892	39.25400
71	004/22	2022-04-21	Domestic animals	Feline	Mjini Magharibi	Mjini	Amani	Negative	-6.16329	39.22016
72	005/22	2022-04-28	Domestic animals	Canine	Kusini Unguja	Kati	Dunga Kiembeni	Negative	-6.15357	39.32970
73	006/22	2022-04-28	Wildlife	Wildlife - Red colobus monkey	Kusini Unguja	Kati	Pete	Negative	-6.26715	39.42843
74	007/22	2022-05-01	Domestic animals	Feline	Mjini Magharibi	Mjini	Mpendae	Negative	-6.18238	39.21543
75	008/22	2022-05-05	Domestic animals	Canine	Mjini Magharibi	Magharibi B	Chukwani	Negative	-6.23333	39.21667
76	009/12	2022-05-25	Domestic animals	Feline	Mjini Magharibi	Mjini	Mikunguni	Negative	-6.16339	39.20715
77	010/22	2022-05-27	Domestic animals	Canine	Kusini Unguja	Kati	Ndijanimseweni	Positive	-6.19012	39.35202
78	011/22	2022-06-12	Domestic animals	Canine	Kusini Unguja	Kati	Ndijanimseweni	Positive	-6.19012	39.35202
79	012/22	2022-06-14	Domestic animals	Feline	Mjini Magharibi	Mjini	Gulioni	Negative	-6.16045	39.20168
80	013/22	2022-06-17	Domestic animals	Canine	Kusini Unguja	Kati	Dunga Bweni	Negative	-6.11512	39.34135

#	Sample number	Date	Binned Species	Species	Province	District	Shehia	Result	Latitude	Longitude
81	014/22	2022-06-28	Domestic animals	Canine	Kusini Unguja	Kusini	Jambiani Kikadini	Negative	-6.32465	39.54590
82	015/22	2022-07-05	Domestic animals	Feline	Mjini Magharibi	Mjini	Mikunguni	Negative	-6.16340	39.20715
83	016/22	2022-07-05	Domestic animals	Canine	Mjini Magharibi	Magharibi A	Dole	Negative	-6.10445	39.25338
84	017/22	2022-07-06	Domestic animals	Feline	Kusini Unguja	Kusini	Paje	Negative	-6.26675	39.53407
85	018/22	2022-07-18	Domestic animals	Canine	Kusini Unguja	Kati	Pagali	Negative	-6.10621	39.34665
86	019/22	2022-08-23	Domestic animals	Canine	Mjini Magharibi	Magharibi B	Uwandani	Negative	-6.20761	39.27341
87	020/22	2022-09-01	Domestic animals	Canine	Mjini Magharibi	Magharibi A	Kibweni	Positive	-6.11466	39.21887
88	021/22	2022-09-15	Domestic animals	Canine	Mjini Magharibi	Magharibi B	Maungani	Positive	-6.23333	39.25000
89	022/22	2022-10-04	Domestic animals	Feline	Mjini Magharibi	Magharibi B	Mwanakwerekwe	Negative	-6.17680	39.23133
90	023/22	2022-10-12	Domestic animals	Feline	Mjini Magharibi	Mjini	Muembeshauri	Negative	-6.16664	39.20122
91	024/22	2022-10-12	Livestock	Caprine	Mjini Magharibi	Magharibi B	Nyamanzi	Negative	-6.26667	39.25000
92	025/22	2022-10-24	Wildlife	Bat	Kusini Unguja	Kati	Dunga Bweni	Negative	-6.11667	39.33333
93	026/22	2022-10-28	Domestic animals	Canine	Kusini Unguja	Kati	Uzini	Negative	-6.07920	39.33554
94	027/22	2022-10-28	Domestic animals	Canine	Kusini Unguja	Kati	Uzini	Negative	-6.07920	39.33554
95	028/22	2022-11-02	Domestic animals	Canine	Mjini Magharibi	Magharibi A	Kijichi	Negative	-6.09891	39.22563
96	029/22	2022-11-03	Domestic animals	Canine	Kusini Unguja	Kati	Ndijanimseweni	Positive	-6.19194	39.34500
97	030/22	2022-11-23	Domestic animals	Feline	Mjini Magharibi	Mjini	Kikwajuni Bondeni	Positive	-6.16672	39.19441
98	031/22	2022-12-04	Domestic animals	Canine	Mjini Magharibi	Mjini	Maruhubi	Negative	-6.14659	39.20969
99	032/22	2022-12-04	Domestic animals	Canine	Mjini Magharibi	Magharibi B	Uwandani	Negative	-6.20285	39.26823
100	033/22	2022-12-24	Domestic animals	Canine	Kusini Unguja	Kati	Ndijanimseweni	Positive	-6.19194	39.34500
101	034/22	2022-12-28	Domestic animals	Canine	Mjini Magharibi	Magharibi B	Maungani	Negative	-6.23333	39.25000
102	035/22	2022-12-28	Domestic animals	Canine	Kusini Unguja	Kati	Bungi	Negative	-6.23899	39.32906
103	036/22	2022-12-28	Domestic animals	Canine	Kaskazini Unguja	Kaskazini A	Banda Kuu	Negative	-5.72560	39.29726
104	001/23	2023-01-09	Domestic animals	Canine	Mjini Magharibi	Magharibi B	Maungani	Negative	-6.23510	39.25055
105	002/23	2023-01-11	Wildlife	Wildlife - Red colobus monkey	Kusini Unguja	Kati	Pete	Negative	-6.27595	39.41635
106	003/23	2023-01-12	Domestic animals	Canine	Kusini Unguja	Kati	Mpapa	Negative	-6.09874	39.33878
107	004/23	2023-01-19	Domestic animals	Canine	Kusini Unguja	Kusini	Jambiani Kikadini	Negative	-6.33390	39.55198
108	006/23	2023-01-25	Wildlife	Wildlife - Vervet monkey	Kusini Unguja	Kati	Tunguu	Positive	-6.20000	39.31667
109	007/23	2023-01-31	Domestic animals	Canine	Mjini Magharibi	Magharibi B	Kisauni	Negative	-6.21411	39.23080

#	Sample number	Date	Binned Species	Species	Province	District	Shehia	Result	Latitude	Longitude
110	008/23	2023-02-01	Livestock	Bovine	Kaskazini Unguja	Kaskazini B	Kilombero	Positive	-6.02435	39.32661
111	009/23	2023-02-10	Domestic animals	Canine	Kaskazini Unguja	Kaskazini B	Kilombero	Positive	-6.02435	39.32661
112	010/23	2023-02-11	Domestic animals	Canine	Mjini Magharibi	Magharibi A	Mtoni	Positive	-6.12644	39.21553
113	011/23	2023-02-14	Domestic animals	Canine	Mjini Magharibi	Magharibi B	Kisauni	Negative	-6.21787	39.23201
114	012/23	2023-02-14	Domestic animals	Canine	Mjini Magharibi	Magharibi B	Uwandani	Positive	-6.20761	39.27341
115	013/23	2023-02-16	Domestic animals	Canine	Mjini Magharibi	Magharibi B	Maungani	Negative	-6.22937	39.24303
116	014/23	2023-02-21	Domestic animals	Canine	Kusini Unguja	Kusini	Jambiani Kibigija	Negative	-6.30556	39.53953
117	015/23	2023-03-20	Domestic animals	Feline	Mjini Magharibi	Magharibi A	Kama	Negative	-6.04694	39.21361
118	016/23	2023-03-21	Domestic animals	Canine	Mjini Magharibi	Magharibi B	Kiembesamaki	Positive	-6.20967	39.20316
119	017/23	2023-03-26	Domestic animals	Canine	Kaskazini Unguja	Kaskazini B	Kilombero	Positive	-6.02021	39.32333
120	018/23	2023-03-28	Domestic animals	Canine	Kusini Unguja	Kati	Jumbi	Positive	-6.20114	39.29741
121	019/23	2023-04-01	Wildlife	Murine/rodent	Mjini Magharibi	Magharibi B	Kiembesamaki	Negative	-6.20387	39.21418
122	020/23	2023-04-03	Domestic animals	Canine	Kusini Unguja	Kusini	Kitogani	Positive	-6.27090	39.42809
123	021/23	2023-04-13	Domestic animals	Canine	Mjini Magharibi	Mjini	Kikwajuni Bondeni	Negative	-6.16550	39.19452
124	022/23	2023-05-04	Domestic animals	Feline	Kusini Unguja	Kati	Unguja Ukuu Kaebona	Negative	-6.28216	39.38378
125	023/23	2023-05-05	Domestic animals	Canine	Kusini Unguja	Kati	Pete	Negative	-6.28835	39.40538
126	024/23	2023-05-15	Domestic animals	Canine	Mjini Magharibi	Magharibi B	Uwandani	Negative	-6.20000	39.26667
127	025/23	2023-05-19	Domestic animals	Feline	Mjini Magharibi	Magharibi B	Pangawe	Negative	-6.18658	39.23917
128	026/23	2023-05-22	Domestic animals	Canine	Mjini Magharibi	Magharibi B	Sokoni	Negative	-6.17734	39.22329
129	027/23	2023-05-24	Domestic animals	Feline	Kaskazini Unguja	Kaskazini B	Kilombero	Negative	-6.01667	39.35000
130	028/23	2023-06-08	Domestic animals	Feline	Kusini Unguja	Kusini	Paje	Positive	-6.27495	39.53419
131	029/23	2023-06-18	Domestic animals	Feline	Kusini Unguja	Kusini	Paje	Negative	-6.27495	39.53419
132	030/23	2023-06-18	Domestic animals	Canine	Mjini Magharibi	Magharibi A	Dole	Negative	-6.10557	39.25364
133	031/23	2023-06-18	Domestic animals	Canine	Mjini Magharibi	Magharibi A	Dole	Negative	-6.10557	39.25364
134	032/23	2023-06-21	Wildlife	Wildlife - Red colobus monkey	Mjini Magharibi	Magharibi A	Dole	Negative	-6.09942	39.26462
135	033/23	2023-06-26	Livestock	Ovine	Mjini Magharibi	Magharibi B	Mombasa	Negative	-6.19580	39.21735
136	034/23	2023-06-27	Domestic animals	Feline	Mjini Magharibi	Magharibi A	Kizimbani	Negative	-6.08333	39.26667
137	035/23	2023-07-16	Livestock	Ovine	Mjini Magharibi	Magharibi A	Kizimbani	Negative	-6.08333	39.26667

#	Sample number	Date	Binned Species	Species	Province	District	Shehia	Result	Latitude	Longitude
138	036/23	2023-07-25	Wildlife	Wildlife - Bush Baby	Mjini Magharibi	Magharibi A	Chuini	Negative	-6.06548	39.22083
139	037/23	2023-07-29	Domestic animals	Feline	Kaskazini Unguja	Kaskazini A	Kigongoni	Negative	-5.82944	39.30222
140	038/23	2023-07-29	Domestic animals	Feline	Mjini Magharibi	Magharibi A	Mtoni	Negative	-6.12581	39.22590
141	039/23	2023-07-30	Wildlife	Wildlife - Vervet monkey	Mjini Magharibi	Mjini	Kiponda	Negative	-6.16037	39.19048
142	040/23	2023-08-29	Domestic animals	Canine	Mjini Magharibi	Magharibi A	Mtonikidatu	Negative	-6.13734	39.22579
143	041/23	2023-08-29	Domestic animals	Canine	Mjini Magharibi	Magharibi A	Mtonikidatu	Negative	-6.13734	39.22579
144	042/23	2023-08-29	Domestic animals	Canine	Mjini Magharibi	Magharibi A	Mtonikidatu	Negative	-6.13734	39.22579
145	043/23	2023-10-17	Wildlife	Wildlife	Kusini Unguja	Kati	Cheju	Negative	-6.21483	39.37872
146	044/23	2023-10-25	Domestic animals	Canine	Mjini Magharibi	Magharibi A	Dole	Negative	-6.09714	39.25199
147	045/23	2023-11-03	Domestic animals	Canine	Mjini Magharibi	Mjini	Maruhubi	Negative	-6.14689	39.21003
148	046/23	2023-11-09	Domestic animals	Feline	Kusini Unguja	Kati	Tindini	Positive	-6.29426	39.36681
149	047/23	2023-11-14	Domestic animals	Canine	Kusini Unguja	Kati	Tindini	Positive	-6.29426	39.36968
150	048/23	2023-12-03	Domestic animals	Canine	Kaskazini Unguja	Kaskazini A	Kilindi	Negative	-5.74408	39.29352
151	001/24	2024-01-13	Domestic animals	Dog	Mjini Magharibi	Magharibi A	Mfenesini	Positive	-6.05003	39.22428
152	002/24	2024-03-02	Livestock	Bovine	Mjini Magharibi	Magharibi A	Mwera	Positive	-6.15796	39.26299
153	003/24	2024-03-02	Domestic animals	Cat	Kusini Unguja	Kati	Uroa	Negative	-6.09794	39.42496
154	004/24	**2024-05-14**	Domestic animals	Dog	Mjini Magharibi	Mjini	Amani	Negative	-6.16329	39.22016
155	005/25	2024-05-20	Domestic animals	Dog	Kaskazini Unguja	Kaskazini B	Kiwengwa	Negative	-5.98544	39.37559
156	006/24	2024-05-21	Domestic animals	Dog	Mjini Magharibi	Magharibi A	Dole	Negative	-6.10445	39.25338
157	007/24	2024-05-22	Domestic animals	Dog	Mjini Magharibi	Magharibi B	Chukwani	Negative	-6.23333	39.21667
158	008/24	2024-07-01	Livestock	Caprine	Mjini Magharibi	Magharibi B	Dimani	Positive	-6.27645	39.27641
159	009/24	2024-07-12	Domestic animals	Cat	Mjini Magharibi	Magharibi A	Dole	Negative	-6.10445	39.25338
160	010/24	2024-07-14	Livestock	Livestock	Mjini Magharibi	Magharibi A	Mfenesini	Negative	-6.04611	39.23222
161	011/24	2024-07-19	Livestock	Caprine	Mjini Magharibi	Magharibi B	Fumba	Positive	-6.30915	39.27764
162	012/24	2024-09-12	Wildlife	Wildlife	Kusini Unguja	Kusini	Kitogani	Negative	-6.27248	39.41895
163	013/24	2024-10-31	Wildlife	Wildlife	Kaskazini Unguja	Kaskazini A	Kilindi	Negative	-5.74408	39.29352
164	014/24	2024-11-01	Domestic animals	Cat	Mjini Magharibi	Mjini	Maruhubi	Negative	-6.14659	39.20969
165	015/24	2024-11-04	Livestock	Livestock	Mjini Magharibi	Magharibi B	Mwanakwerekwe	Negative	-6.17680	39.23133

Table S4: Dog density (dogs/km²) for all shehias in Unguja Island.

Shehia	Estimated dog population[1]	Dog density	Threshold	Calculated dog density	Threshold	New Estimated Dog population	Original/ estimated value
Amani	Unknown	Unknown	Unknown	58.644	Above	17.17	Estimated
Bambi	109	4.721	Above	4.721	Above	109.00	Original
Banda Kuu	29	15.838	Above	15.838	Above	29.00	Original
Bandamaji	0	0.000	Below	0.000	Below	0.00	Original
Banko	Unknown	Unknown	Unknown	41.405	Above	19.28	Estimated
Binguni	68	7.922	Above	7.922	Above	68.00	Original
Bububu	147	166.440	Above	166.440	Above	147.00	Original
Bumbwisudi	100	12.442	Above	12.442	Above	100.00	Original
Bungi	45	3.363	Above	3.363	Above	45.00	Original
Bwejuu	62	4.651	Above	4.651	Above	62.00	Original
Bweleo	7	0.707	Below	0.707	Below	7.00	Original
Bwereu	0	0.000	Below	0.000	Below	0.00	Original
Chaani Kubwa	318	89.502	Above	89.502	Above	318.00	Original
Chaani Masingini	5	0.700	Below	0.700	Below	5.00	Original
Charawe	36	1.118	Below	1.118	Below	36.00	Original
Cheju	93	3.317	Above	3.317	Above	93.00	Original
Chemchem	Unknown	Unknown	Unknown	26.452	Above	35.53	Estimated
Chuini	89	15.201	Above	15.201	Above	89.00	Original
Chukwani	37	5.891	Above	5.891	Above	37.00	Original
Chumbuni	Unknown	Unknown	Unknown	45.150	Above	20.29	Estimated
Chunga	Unknown	Unknown	Unknown	10.952	Above	50.15	Estimated
Chutama	0	0.000	Below	0.000	Below	0.00	Original
Chwaka	39	3.054	Above	3.054	Above	39.00	Original
Dimani	24	3.009	Above	3.009	Above	24.00	Original
Dole	50	5.952	Above	5.952	Above	50.00	Original
Donge Karange	36	3.728	Above	3.728	Above	36.00	Original
Donge Mbiji	28	8.969	Above	8.969	Above	28.00	Original
Donge Mtambile	39	26.351	Above	26.351	Above	39.00	Original
Donge Pwani	Unknown	Unknown	Unknown	3.767	Above	9.55	Estimated
Donge Vijibweni	48	91.151	Above	91.151	Above	48.00	Original
Dongwe	9	0.288	Below	0.288	Below	9.00	Original
Dunga Bweni	174	11.395	Above	11.395	Above	174.00	Original
Dunga Kiembeni	222	20.163	Above	20.163	Above	222.00	Original
Fujoni	114	22.687	Above	22.687	Above	114.00	Original
Fukuchani	17	6.714	Above	6.714	Above	17.00	Original
Fumba	30	7.564	Above	7.564	Above	30.00	Original
Fuoni Kipungani	Unknown	Unknown	Unknown	15.201	Above	36.36	Estimated
Fuoni Migombani	Unknown	Unknown	Unknown	21.708	Above	19.75	Estimated
Gamba	41	12.885	Above	12.885	Above	41.00	Original
Ghana	91	12.056	Above	12.056	Above	91.00	Original

[1] Information about dog population numbers were obtained from the 2022 GARC technical report found on:
14. GARC. *Zanzibar*. 2025 November 2025]; Available from: https://rabiesalliance.org/country/zanzibar,
15. Coetzer, A., *Updating the estimated dog population in Zanzibar using multi-year dog vaccination data tracked using the Rabies Vaccination Tracker (RVT) tool. Technical Report, 2022*. 2022.

Shehia	Estimated dog population[1]	Dog density	Threshold	Calculated dog density	Threshold	New Estimated Dog population	Original/ estimated value
Gomani	Unknown	Unknown	Unknown	1.406	Above	5.58	Estimated
Gulioni	Unknown	Unknown	Unknown	49.113	Above	6.58	Estimated
Hawaii	Unknown	Unknown	Unknown	4.882	Above	9.42	Estimated
Jambiani Kibigija	80	2.815	Above	2.815	Above	80.00	Original
Jambiani Kikadini	43	1.015	Below	1.015	Below	43.00	Original
Jang'ombe	Unknown	Unknown	Unknown	51.141	Above	16.54	Estimated
Jendele	68	5.960	Above	5.960	Above	68.00	Original
Jitimai	Unknown	Unknown	Unknown	25.204	Above	10.41	Estimated
Jongowe	Unknown	Unknown	Unknown	3.819	Above	19.14	Estimated
Juga Kuu	Unknown	Unknown	Unknown	1.617	Above	10.18	Estimated
Jumbi	98	5.879	Above	5.879	Above	98.00	Original
Kajengwa	38	1.337	Below	1.337	Below	38.00	Original
Kama	30	5.473	Above	5.473	Above	30.00	Original
Kandwi	49	2.993	Above	2.993	Above	49.00	Original
Karakana	Unknown	Unknown	Unknown	29.617	Above	6.87	Estimated
Kianga	92	10.827	Above	10.827	Above	92.00	Original
Kibeni	0	0.000	Below	0.000	Below	0.00	Original
Kiboje Mkwajuni	68	9.214	Above	9.214	Above	68.00	Original
Kiboje Mwembeshauri	50	5.260	Above	5.260	Above	50.00	Original
Kibondeni	257	24.735	Above	24.735	Above	257.00	Original
Kibuteni	22	1.135	Below	1.135	Below	22.00	Original
Kibweni	16	19.050	Above	19.050	Above	16.00	Original
Kidanzini	1	0.130	Below	0.130	Below	1.00	Original
Kidimni	117	13.994	Above	13.994	Above	117.00	Original
Kidombo	4	1.459	Above	1.459	Above	4.00	Original
Kidongo Chekundu	44	558.943	Above	558.943	Above	44.00	Original
Kidoti	53	4.118	Above	4.118	Above	53.00	Original
Kiembesamaki	7	4.403	Above	4.403	Above	7.00	Original
Kigomani	Unknown	Unknown	Unknown	2.990	Above	18.78	Estimated
Kigongoni	Unknown	Unknown	Unknown	1.436	Above	8.19	Estimated
Kigunda	27	11.749	Above	11.749	Above	27.00	Original
Kihinani	46	24.973	Above	24.973	Above	46.00	Original
Kijibwemtu	Unknown	Unknown	Unknown	0.170	Below	1.91	Estimated
Kijichi	Unknown	Unknown	Unknown	20.417	Above	40.75	Estimated
Kijini (Kusini)	34	2.838	Above	2.838	Above	34.00	Original
Kijini Matemwe (Kaskazini A)	55	3.284	Above	3.284	Above	55.00	Original
Kijitoupele	21	8.099	Above	8.099	Above	21.00	Original
Kikaangoni	Unknown	Unknown	Unknown	29.105	Above	37.87	Estimated
Kikobweni	24	4.985	Above	4.985	Above	24.00	Original
Kikungwi	39	5.052	Above	5.052	Above	39.00	Original
Kikwajuni Bondeni	Unknown	Unknown	Unknown	47.259	Above	6.74	Estimated
Kikwajuni Juu	Unknown	Unknown	Unknown	16.296	Above	6.68	Estimated
Kilimahewa Bondeni	Unknown	Unknown	Unknown	86.956	Above	14.83	Estimated
Kilimahewa Juu	Unknown	Unknown	Unknown	64.460	Above	13.32	Estimated
Kilimani (Mijini)	Unknown	Unknown	Unknown	9.590	Above	6.56	Estimated
Kilimani Tazari (Kaskazini A)	3	0.591	Below	0.591	Below	3.00	Original

Shehia	Estimated dog population[1]	Dog density	Threshold	Calculated dog density	Threshold	New Estimated Dog population	Original/ estimated value
Kilindi	3	0.183	Below	0.183	Below	3.00	Original
Kilombero	38	2.350	Above	2.350	Above	38.00	Original
Kinduni	32	5.127	Above	5.127	Above	32.00	Original
Kinuni	18	32.775	Above	32.775	Above	18.00	Original
Kinyasini	291	58.317	Above	58.317	Above	291.00	Original
Kiombamvua	18	4.324	Above	4.324	Above	18.00	Original
Kiongoni	8	6.536	Above	6.536	Above	8.00	Original
Kiongwe Kidogo	Unknown	Unknown	Unknown	0.974	Below	6.45	Estimated
Kipange	22	3.114	Above	3.114	Above	22.00	Original
Kiponda	Unknown	Unknown	Unknown	43.689	Above	3.80	Estimated
Kisauni	96	15.301	Above	15.301	Above	96.00	Original
Kisimamajongoo	Unknown	Unknown	Unknown	89.339	Above	6.48	Estimated
Kisiwandui	Unknown	Unknown	Unknown	43.599	Above	3.77	Estimated
Kisongoni	86	41.687	Above	41.687	Above	86.00	Original
Kitogani	33	2.072	Above	2.072	Above	33.00	Original
Kitope	19	3.156	Above	3.156	Above	19.00	Original
Kiungani	Unknown	Unknown	Unknown	9.263	Above	28.93	Estimated
Kivunge	96	81.425	Above	81.425	Above	96.00	Original
Kiwengwa	93	3.864	Above	3.864	Above	93.00	Original
Kizimbani	57	6.897	Above	6.897	Above	57.00	Original
Kizimkazi Dimbani	11	0.940	Below	0.940	Below	11.00	Original
Kizimkazi Mkunguni	12	1.013	Below	1.013	Below	12.00	Original
Koani	97	13.595	Above	13.595	Above	97.00	Original
Kombeni	25	2.404	Above	2.404	Above	25.00	Original
Kwa Goa	Unknown	Unknown	Unknown	19.676	Above	14.94	Estimated
Kwa Mchina	Unknown	Unknown	Unknown	15.871	Above	10.17	Estimated
Kwa Wazee	Unknown	Unknown	Unknown	30.250	Above	12.60	Estimated
Kwaalimsha	Unknown	Unknown	Unknown	64.347	Above	7.81	Estimated
Kwaalinatu	Unknown	Unknown	Unknown	56.060	Above	9.54	Estimated
Kwabintiamrani	Unknown	Unknown	Unknown	55.319	Above	24.77	Estimated
Kwagube	Unknown	Unknown	Unknown	8.586	Above	24.23	Estimated
Kwahani	Unknown	Unknown	Unknown	72.995	Above	13.27	Estimated
Kwamtipura	Unknown	Unknown	Unknown	83.245	Above	23.98	Estimated
Kwamtumwajeni	Unknown	Unknown	Unknown	64.717	Above	10.87	Estimated
Machui	20	5.049	Above	5.049	Above	20.00	Original
Mafufuni	22	1.366	Above	1.366	Above	22.00	Original
Magogoni	25	39.240	Above	39.240	Above	25.00	Original
Magomeni	Unknown	Unknown	Unknown	43.477	Above	18.26	Estimated
Mahonda	59	15.963	Above	15.963	Above	59.00	Original
Majenzi	Unknown	Unknown	Unknown	0.985	Below	4.85	Estimated
Makadara	Unknown	Unknown	Unknown	75.399	Above	6.32	Estimated
Makoba	6	1.190	Below	1.190	Below	6.00	Original
Malindi	Unknown	Unknown	Unknown	21.403	Above	8.58	Estimated
Mambosasa	Unknown	Unknown	Unknown	23.914	Above	48.76	Estimated
Mangapwani	40	7.360	Above	7.360	Above	40.00	Original
Mapinduzi	Unknown	Unknown	Unknown	52.588	Above	6.62	Estimated

Shehia	Estimated dog population[1]	Dog density	Threshold	Calculated dog density	Threshold	New Estimated Dog population	Original/ estimated value
Maruhubi	Unknown	Unknown	Unknown	13.101	Above	10.38	Estimated
Marumbi	45	3.753	Above	3.753	Above	45.00	Original
Masingini	Unknown	Unknown	Unknown	7.601	Above	37.63	Estimated
Masumbani	Unknown	Unknown	Unknown	79.716	Above	14.01	Estimated
Matarumbeta	Unknown	Unknown	Unknown	29.648	Above	12.37	Estimated
Matemwe Kaskazini	100	22.681	Above	22.681	Above	100.00	Original
Matemwe Kusini	Unknown	Unknown	Unknown	1.948	Above	30.82	Estimated
Matetema	24	5.142	Above	5.142	Above	24.00	Original
Maungani	93	15.911	Above	15.911	Above	93.00	Original
Mbaleni	104	10.582	Above	10.582	Above	104.00	Original
Mboriborini	Unknown	Unknown	Unknown	95.910	Above	7.19	Estimated
Mbuyutende	Unknown	Unknown	Unknown	1.630	Above	13.99	Estimated
Mbuzini	27	3.389	Above	3.389	Above	27.00	Original
Mbweni	Unknown	Unknown	Unknown	13.879	Above	25.73	Estimated
Mchangani (Kaskazini A)	33	9.857	Above	9.857	Above	33.00	Original
Mchangani (Mjini)	Unknown	Unknown	Unknown	39.453	Above	5.78	Estimated
Mchangani Shamba (Kati)	74	7.334	Above	7.334	Above	74.00	Original
Mchenza Shauri	2	1.705	Above	1.705	Above	2.00	Original
Melinne	45	83.195	Above	83.195	Above	45.00	Original
Meya	Unknown	Unknown	Unknown	75.232	Above	16.96	Estimated
Mfenesini	13	4.100	Above	4.100	Above	13.00	Original
Mgambo	129	12.874	Above	12.874	Above	129.00	Original
Mgeni Haji	119	38.227	Above	38.227	Above	119.00	Original
Michamvi	22	1.549	Above	1.549	Above	22.00	Original
Michikichini	Unknown	Unknown	Unknown	27.528	Above	55.99	Estimated
Michungwani	Unknown	Unknown	Unknown	17.659	Above	21.77	Estimated
Miembeni	Unknown	Unknown	Unknown	73.077	Above	10.55	Estimated
Migombani	Unknown	Unknown	Unknown	14.302	Above	22.24	Estimated
Mikarafuuni	Unknown	Unknown	Unknown	34.340	Above	23.57	Estimated
Mikunguni	Unknown	Unknown	Unknown	50.790	Above	7.86	Estimated
Misufini	23	2.787	Above	2.787	Above	23.00	Original
Mitakawani	13	6.900	Above	6.900	Above	13.00	Original
Mitiulaya	Unknown	Unknown	Unknown	33.525	Above	7.94	Estimated
Miwani	59	9.180	Above	9.180	Above	59.00	Original
Mkadini	45	18.435	Above	18.435	Above	45.00	Original
Mkataleni	51	7.338	Above	7.338	Above	51.00	Original
Mkele	Unknown	Unknown	Unknown	78.949	Above	11.69	Estimated
Mkokotoni	39	10.234	Above	10.234	Above	39.00	Original
Mkunazini	Unknown	Unknown	Unknown	38.473	Above	4.64	Estimated
Mkwajuni	53	23.195	Above	23.195	Above	53.00	Original
Mlandege	Unknown	Unknown	Unknown	51.081	Above	5.23	Estimated
Mnarani	Unknown	Unknown	Unknown	35.152	Above	43.03	Estimated
Mnazimmoja	Unknown	Unknown	Unknown	22.722	Above	5.98	Estimated
Mnyimbi	30	5.293	Above	5.293	Above	30.00	Original
Moga	2	0.780	Below	0.780	Below	2.00	Original
Mombasa	51	63.049	Above	63.049	Above	51.00	Original

Shehia	Estimated dog population[1]	Dog density	Threshold	Calculated dog density	Threshold	New Estimated Dog population	Original/ estimated value
Mpapa	26	23.788	Above	23.788	Above	26.00	Original
Mpendae	23	38.858	Above	38.858	Above	23.00	Original
Mtakuja	Unknown	Unknown	Unknown	3.245	Above	13.73	Estimated
Mtende	Unknown	Unknown	Unknown	0.436	Below	8.73	Estimated
Mto pepo	58	68.396	Above	68.396	Above	58.00	Original
Mto wa Pwani	3	0.616	Below	0.616	Below	3.00	Original
Mtofaani	48	44.902	Above	44.902	Above	48.00	Original
Mtoni	17	4.439	Above	4.439	Above	17.00	Original
Mtoni ChemChem	Unknown	Unknown	Unknown	41.650	Above	59.39	Estimated
Mtoni Kidatu	77	64.924	Above	64.924	Above	77.00	Original
Muembe Majogoo	Unknown	Unknown	Unknown	67.210	Above	31.25	Estimated
Muembe Mchomeke	Unknown	Unknown	Unknown	19.744	Above	32.78	Estimated
Muembeshauri	Unknown	Unknown	Unknown	48.492	Above	5.20	Estimated
Muembetanga	Unknown	Unknown	Unknown	78.160	Above	6.67	Estimated
Munduli	Unknown	Unknown	Unknown	32.951	Above	32.82	Estimated
Muungano	Unknown	Unknown	Unknown	58.550	Above	13.75	Estimated
Muungoni	26	1.143	Below	1.143	Below	26.00	Original
Muwanda	33	6.524	Above	6.524	Above	33.00	Original
Muwange	119	74.515	Above	74.515	Above	119.00	Original
Muyuni A	35	2.371	Above	2.371	Above	35.00	Original
Muyuni B	5	0.484	Below	0.484	Below	5.00	Original
Muyuni C	29	1.655	Above	1.655	Above	29.00	Original
Mwakaje	67	6.449	Above	6.449	Above	67.00	Original
Mwanakwerekwe	56	56.916	Above	56.916	Above	56.00	Original
Mwanyanya	35	23.569	Above	23.569	Above	35.00	Original
Mwembeladu	Unknown	Unknown	Unknown	49.793	Above	7.12	Estimated
MwembeMadema	Unknown	Unknown	Unknown	14.505	Above	5.16	Estimated
Mwembemakumbi	Unknown	Unknown	Unknown	65.395	Above	13.26	Estimated
Mwera	132	20.021	Above	20.021	Above	132.00	Original
Mzuri	14	3.544	Above	3.544	Above	14.00	Original
Ndijani Mseweni	93	9.020	Above	9.020	Above	93.00	Original
Ndijani Mwembepunda	51	10.149	Above	10.149	Above	51.00	Original
Ng'ambwa	32	2.700	Above	2.700	Above	32.00	Original
Nganani	29	3.247	Above	3.247	Above	29.00	Original
Njia ya Mtoni	39	8.238	Above	8.238	Above	39.00	Original
Nyamanzi	54	30.303	Above	30.303	Above	54.00	Original
Nyerere	Unknown	Unknown	Unknown	77.703	Above	18.13	Estimated
Pagali	59	4.525	Above	4.525	Above	59.00	Original
Paje	78	2.237	Above	2.237	Above	78.00	Original
Pale	16	1.884	Above	1.884	Above	16.00	Original
Pangawe	88	110.901	Above	110.901	Above	88.00	Original
Pangeni	34	4.545	Above	4.545	Above	34.00	Original
Pete	44	1.882	Above	1.882	Above	44.00	Original
Pitanazako	4	2.404	Above	2.404	Above	4.00	Original
Pongwe	44	2.895	Above	2.895	Above	44.00	Original
Potoa	20	4.006	Above	4.006	Above	20.00	Original

Shehia	Estimated dog population[1]	Dog density	Threshold	Calculated dog density	Threshold	New Estimated Dog population	Original/ estimated value
Pwani Mchangani	30	4.422	Above	4.422	Above	30.00	Original
Rahaleo	Unknown	Unknown	Unknown	45.670	Above	5.98	Estimated
Saateni	Unknown	Unknown	Unknown	23.094	Above	7.18	Estimated
Sebleni	Unknown	Unknown	Unknown	54.287	Above	19.70	Estimated
Shakani	32	4.268	Above	4.268	Above	32.00	Original
Shangani	Unknown	Unknown	Unknown	29.989	Above	5.20	Estimated
Sharifu Msa	8	8.497	Above	8.497	Above	8.00	Original
Shaurimoyo	Unknown	Unknown	Unknown	71.470	Above	15.72	Estimated
Sogea	Unknown	Unknown	Unknown	78.553	Above	13.23	Estimated
Sokoni	Unknown	Unknown	Unknown	21.785	Above	13.66	Estimated
Tasani	4	1.157	Below	1.157	Below	4.00	Original
Taveta	Unknown	Unknown	Unknown	44.876	Above	15.24	Estimated
Tazari	30	4.950	Above	4.950	Above	30.00	Original
Tindini	65	11.533	Above	11.533	Above	65.00	Original
Tomondo	33	26.807	Above	26.807	Above	33.00	Original
Tunduni	23	8.785	Above	8.785	Above	23.00	Original
Tunguu	51	2.963	Above	2.963	Above	51.00	Original
Ubago	86	6.413	Above	6.413	Above	86.00	Original
Uholanzi	Unknown	Unknown	Unknown	61.062	Above	22.87	Estimated
Ukongoroni	64	1.847	Above	1.847	Above	64.00	Original
Umbuji	126	4.201	Above	4.201	Above	126.00	Original
Unguja Ukuu Kaebona	55	4.872	Above	4.872	Above	55.00	Original
Unguja Ukuu Kaepwani	66	15.896	Above	15.896	Above	66.00	Original
Upenja	11	0.562	Below	0.562	Below	11.00	Original
Uroa	13	0.729	Below	0.729	Below	13.00	Original
Urusi	Unknown	Unknown	Unknown	68.528	Above	20.68	Estimated
Uvivini	Unknown	Unknown	Unknown	5.167	Above	21.44	Estimated
Uwandani	Unknown	Unknown	Unknown	5.132	Above	51.31	Estimated
Uzi (Kati)	36	9.769	Above	9.769	Above	36.00	Original
Uzi (Magharibi B)	Unknown	Unknown	Unknown	46.741	Above	50.53	Estimated
Uzini	25	9.608	Above	9.608	Above	25.00	Original
Vikokotoni	Unknown	Unknown	Unknown	35.445	Above	4.54	Estimated
Welezo	17	21.662	Above	21.662	Above	17.00	Original
Zawiyani	Unknown	Unknown	Unknown	0.229	Below	4.98	Estimated
Zingwezingwe	27	4.184	Above	4.184	Above	27.00	Original
				**Total dog population**		**9865.01**	

References

1. Troupin, C., L. Dacheux, M. Tanguy, C. Sabeta, H. Blanc, C. Bouchier, M. Vignuzzi, S. Duchene, E.C. Holmes, and H. Bourhy, *Large-scale phylogenomic analysis reveals the complex evolutionary history of rabies virus in multiple carnivore hosts.* PLoS pathogens, 2016. **12**(12): p. e1006041. DOI: https://doi.org/10.1371/journal.ppat.1006041.
2. Johnson, N., M. Letshwenyo, E. Baipoledi, G. Thobokwe, and A. Fooks, *Molecular epidemiology of rabies in Botswana: a comparison between antibody typing and nucleotide sequence phylogeny.* Veterinary microbiology, 2004. **101**(1): p. 31-38.
3. Sabeta, C.T., D.A. Marston, L.M. McElhinney, D.L. Horton, B.M. Phahladira, and A.R. Fooks, *Rabies in the African civet: an incidental host for lyssaviruses?* Viruses, 2020. **12**(4): p. 368. DOI: https://doi.org/10.3390/v12040368.
4. Kgaladi, J., L.H. Nel, and W. Markotter, *Comparison of pathogenic domains of rabies and African rabies-related lyssaviruses and pathogenicity observed in mice.* Onderstepoort Journal of Veterinary Research, 2013. **80**(1): p. 1-13.
5. Brunker, K., D.A. Marston, D.L. Horton, S. Cleaveland, A.R. Fooks, R. Kazwala, C. Ngeleja, T. Lembo, M. Sambo, and Z.J. Mtema, *Elucidating the phylodynamics of endemic rabies virus in eastern Africa using whole-genome sequencing.* Virus evolution, 2015. **1**(1): p. vev011. DOI: https://doi.org/10.1093/ve/vev011.
6. Lushasi, K., K. Brunker, M. Rajeev, E.A. Ferguson, G. Jaswant, L.L. Baker, R. Biek, J. Changalucha, S. Cleaveland, and A. Czupryna, *Integrating contact tracing and whole-genome sequencing to track the elimination of dog-mediated rabies: an observational and genomic study.* Elife, 2023. **12**: p. e85262. DOI: https://doi.org/10.7554/eLife.85262.
7. Mapatse, M., E. Ngoepe, D. Abernethy, J.M. Fafetine, I. Anahory, and C. Sabeta, *Rabies Virus Seroprevalence among Dogs in Limpopo National Park and the Phylogenetic Analyses of Rabies Viruses in Mozambique.* Pathogens, 2022. **11**(9): p. 1043. DOI: https://doi.org/10.3390/pathogens11091043.
8. Malan, A.J., A. Coetzer, C.T. Sabeta, and L.H. Nel, *Epidemiological interface of sylvatic and dog rabies in the North West Province of South Africa.* Tropical Medicine and Infectious Disease, 2022. **7**(6): p. 90. DOI: https://doi.org/10.3390/tropicalmed7060090.
9. Jaswant, G., K. Campbell, A. Czupryna, A. Mwatondo, B. Ogoti, C.W. Embregts, C.H. GeurtsvanKessel, C. Kayuki, D. Kuchaka, and G. Wambura, *Molecular characterisation of human rabies in Tanzania and Kenya: a case series report and phylogenetic investigation.* Infectious diseases of poverty, 2024. **13**(1): p. 79. DOI: https://doi.org/10.1186/s40249-024-01245-w.
10. Castellan, M., G. Zamperin, G. Foiani, M. Zorzan, M.F. Priore, P. Drzewnioková, E. Melchiotti, M. Vascellari, I. Monne, and S. Crovella, *Immunological findings of West Caucasian bat virus in an accidental host.* Journal of Virology, 2025. **99**(2): p. e01914-24. DOI: https://doi.org/10.1128/jvi.01914-24.
11. Marston, D., L. McElhinney, Y. Ali, K. Intisar, S. Ho, C. Freuling, T. Müller, and A. Fooks, *Phylogenetic analysis of rabies viruses from Sudan provides evidence of a viral clade with a unique molecular signature.* Virus research, 2009. **145**(2): p. 244-250. DOI: https://doi.org/10.1016/j.virusres.2009.07.010.
12. Johnson, N., L. McElhinney, Y. Ali, I.K. Saeed, and A. Fooks, *Molecular epidemiology of canid rabies in Sudan: evidence for a common origin of rabies with Ethiopia.* Virus research, 2004. **104**(2): p. 201-205.
13. Tordo, N., O. Poch, A. Ermine, G. Keith, and F. Rougeon, *Completion of the rabies virus genome sequence determination: highly conserved domains among the L (polymerase) proteins of unsegmented negative-strand RNA viruses.* Virology, 1988. **165**(2): p. 565-576.
14. GARC. *Zanzibar*. 2025 November 2025]; Available from: https://rabiesalliance.org/country/zanzibar.
15. Coetzer, A., *Updating the estimated dog population in Zanzibar using multi-year dog vaccination data tracked using the Rabies Vaccination Tracker (RVT) tool. Technical Report, 2022*. 2022.
